# Knowledge, attitudes, beliefs, and stigma related to latent tuberculosis infection: a qualitative study among Eritreans in the Netherlands

**DOI:** 10.1186/s12889-020-09697-z

**Published:** 2020-10-23

**Authors:** Ineke Spruijt, Dawit Tesfay Haile, Susan van den Hof, Kathy Fiekert, Niesje Jansen, Degu Jerene, Eveline Klinkenberg, Ieva Leimane, Jeanine Suurmond

**Affiliations:** 1 KNCV Tuberculosis Foundation, Maanweg 174, 2516 AB The Hague, The Netherlands; 2 Department of Public Health, Amsterdam Public Health Research Institute, Amsterdam University Medical Centre, University of Amsterdam, Meibergdreef 9, 1105 AZ Amsterdam, The Netherlands; 3 Present Address: National Institute for Public Health and the Environment (RIVM), Centre for Infectious Disease Control, Bilthoven, The Netherlands; 4 Department of Global Health and Amsterdam Institute for Global Health and Development, Amsterdam University Medical Centres, Amsterdam, The Netherlands

**Keywords:** Latent tuberculosis infection, Stigma, Tuberculosis prevention, Asylum seekers, Refugees, Low incidence country

## Abstract

**Background:**

Tailored and culturally appropriate latent tuberculosis (TB) infection screening and treatment programs, including interventions against TB stigma, are needed to reduce TB incidence in low TB incidence countries. However, we lack insights in stigma related to latent TB infection (LTBI) among target groups, such as asylum seekers and refugees. We therefore studied knowledge, attitudes, beliefs, and stigma associated with LTBI among Eritrean asylum seekers and refugees in the Netherlands.

**Methods:**

We used convenience sampling to interview adult Eritrean asylum seekers and refugees: 26 semi-structured group interviews following TB and LTBI related health education and LTBI screening, and 31 semi-structured individual interviews with Eritreans during or after completion of LTBI treatment (November 2016–May 2018). We used a thematic analysis to identify, analyse and report patterns in the data.

**Results:**

Despite TB/LTBI education, misconceptions embedded in cultural beliefs about TB transmission and prevention persisted. Fear of getting infected with TB was the cause of reported enacted (isolation and gossip) and anticipated (concealment of treatment and self-isolation) stigma by participants on LTBI treatment.

**Conclusion:**

The inability to differentiate LTBI from TB disease and consequent fear of getting infected by persons with LTBI led to enacted and anticipated stigma comparable to stigma related to TB disease among Eritreans. Additional to continuous culturally sensitive education activities, TB prevention programs should implement evidence-based interventions reducing stigma at all phases in the LTBI screening and treatment cascade.

**Supplementary information:**

**Supplementary information** accompanies this paper at 10.1186/s12889-020-09697-z.

## Background

Low tuberculosis (TB) incidence countries focus on TB prevention through latent TB infection (LTBI) screening and treatment programs, which are tailored to specific populations of interest with a high risk to develop TB, such as immigrants and asylum seekers from high TB incidence countries [1–4]. Although policies between European Union countries differ with regards to type of entry screening (pre or post), and whom is to be screened in terms of age, TB incidence in the country of origin, and reason for immigration, more than half of the European countries have implemented LTBI screening for migrants [5, 6]. Sixteen out of 36 European countries with a low and intermediate TB incidence reported systematic LTBI screening of refugees, whereas four countries reported systematic screening among other migrants (such as labour migrants) [6].

In the Netherlands, a low TB incidence country, 74% of TB patients in 2018 were foreign-born, of which 58% were asylum seekers or refugees living in the Netherlands for less than five years [7]. More particularly, TB burden in the Netherlands is currently highest among Eritrean asylum seekers and refugees with TB incidence rates of 925/100,000 person-years in the first year and 309/100,000 person-years in the fifth year post arrival [8]. Following the need to increase efforts to reduce the TB incidence and the positive results of three implementation studies on LTBI screening and treatment among high TB risk migrants groups [9–11], Dutch policy advisors are now debating about expanding the implementation of post-arrival LTBI screening to other subgroups of immigrants and asylum seekers [4].

Stigma can hamper the effectiveness of LTBI screening and treatment programs as it may impede the uptake of testing and treatment because of people’s concern about reputational loss and stigma by participating in a “TB project” [12]. There is abundant evidence showing that insufficient knowledge about TB and negative social attitudes towards TB is related to stigmatization [13]. Consequently, patients conceal their disease, do not comply with treatment, and isolate themselves. It is therefore important to address LTBI related stigma [14, 15]. However, we lack insights in the burden of LTBI related stigma among target groups for TB prevention activities in low TB incidence countries [16, 17].

Scambler (2009) defined stigma as “a social process, experienced or anticipated, characterized by exclusion, rejection, blame or devaluation that results from experience, perception or reasonable anticipation of an adverse social judgement about a person or a group.” [18] Stigma can be classified in three categories [16, 19] [1]: enacted stigma, in which a person experiences exclusion and/or discrimination. For example, TB patients are regularly rejected by family, friends or communities, resulting from fear of TB [20] [2].; felt stigma, in which a person has feelings of shame, fear, and guilt regarding their (potentially) stigmatized disease. For example, labelling immigrants as high-TB-risk groups can induce feelings of discrimination [20] [3]. anticipated stigma, in which a person perceives, fears and/or expects stigmatization because of their disease. For example, Chinese immigrants in Canada believed that society would exclude those with an LTBI diagnosis [17]. Out of fear of exclusion, they would conceal their LTBI diagnosis and treatment to people in their social network. Besides suffering of the individual, stigma can also negatively affect public health: fear of TB and related stigma can cause diagnostic and treatment delays, as persons would rather hide their symptoms than seek care, leading to a continuous risk of TB transmission, more severe morbidity, and poorer treatment outcomes [19–21]. Furthermore, concealment can lead to continuous risk behaviour and failure to embrace preventive measures [19].

Despite worldwide increasing efforts to rapidly implement TB prevention activities among target groups such as high-TB-risk migrants, very few studies have focused on LTBI knowledge, attitudes, beliefs, and stigma. We therefore studied LTBI related knowledge, attitudes, beliefs, and stigma among Eritrean asylum seekers and refugees -currently the largest group of people with TB disease in the Netherlands [8, 22].

## Methods

This qualitative study was part of the TB-ENDPoint project, which studied the implementation, national impact, and cost effectiveness of LTBI screening and treatment among high-TB-risk migrant groups in the Netherlands. This project incorporated three mixed method studies (qualitative and quantitative research methods), which evaluated the implementation of LTBI screening and treatment among 1) immigrants [9], 2) asylum seekers -predominantly Eritreans- living in asylum seeker centres [11], and 3) Eritrean refugees living in communities for maximum ten years [10]. Additional file 1 provides a detailed description of the TB ENDPoint project and definitions of the high TB risk migrant groups (See Additional file 1). As part of the LTBI screening, participants received TB and LTBI group education before LTBI testing. During the education session, participants learned about tuberculosis (cause, symptoms, transmission, treatment, and global and Dutch epidemiology) and LTBI (what is LTBI, prevention of tuberculosis, importance and aim of LTBI screening and treatment). The education session was designed in an interactive manner and consisted of a presentation using posters or PowerPoint slides. The education, including the posters and PowerPoint slides, was taught in the participants’ mother tongue, using professional interpreters. Furthermore, participants with an LTBI diagnosis received further education by the TB physicians and nurses throughout the care cascade, also with use of professional interpreters. Additional file 2 gives an overview of the content of the TB and LTBI education session (See Additional file 2).

### Study setting and population

For this paper we used data from semi-structured interviews with Eritrean asylum seekers living in asylum seeker centres and Eritrean refugees living in communities in the Netherlands. We applied a convenience sampling strategy because the population of interest -Eritrean asylum seekers and refugees- were accessible at the time of screening and during treatment [23]. Furthermore, the study’s Eritrean research assistant (DTH) was able to earn trust and respect of the participants at the site of screening, which was crucial for the population to agree to participate in the interviews. We invited adult Eritreans present at the LTBI screening to participate in a group interview, which took place immediately following the screening in a separate room, to allow for more privacy and a quiet environment, at the screening site. Additionally, TB nurses asked participants on LTBI treatment for written permission to be approached by phone by DTH to be invited to participate in an individual interview. After consent, DTH set an appointment and planned the individual interview on a location to the participant’s convenience.

Eritreans from both studies were predominantly from the same cohort (arrival in The Netherlands 2013–2017) of Eritrean asylum seekers: the majority of these Eritreans are young (majority between 14 and 30 years of age), male (60%), from the ethnic group Tigrinya and member of the Eritrean Orthodox church (90%), literate but with primary or secondary education, and from rural areas [24].

### Data collection

All interviews primarily focused on identifying facilitators and barriers for LTBI screening and treatment. The interview topic guide contained sections focused on TB background knowledge, TB and LTBI knowledge acquired during pre-LTBI screening education, attitudes, and beliefs about TB, and experienced or perceived TB and LTBI related stigma. Those interview sections were the source of the data used in this study. Interview topic guides were reviewed by the researcher (IS), senior researcher (JS), and DTH. Additional file 3 provides the interview topic guides used for interviews among Eritrean asylum seekers (chapter 1) and refugees (chapter 2) (See additional file 3). Interviews with Eritrean asylum seekers (November 2016–December 2017) and Eritrean refugees (January 2018–May 2018) were conducted by DTH after receiving training by IS and JS on conducting semi-structured interviews. We aimed to minimize interview bias by providing this training and discussing the interviews afterwards with DTH, IS and JS. Interview bias still may have occurred. The interviewer recalled a few missed opportunities for follow-up questions and elaborations during interviews – an insider bias. However, the fact that DTH has a similar background as the interview participants also meant that he knew how to probe further on important topics.

In total, we conducted 26 semi-structured group interviews (21 with Eritrean asylum seekers and 5 with Eritrean refugees) with 2–12 participants (duration 30–60 min) directly after the LTBI education and screening, in a separate room to ensure privacy. Additionally, we conducted 31 individual interviews (21 with Eritrean asylum seekers and 10 with Eritrean refugees) (duration 15–60 min) with participants who were on, or recently completed, LTBI treatment. All participants received information about the study. In an information letter, the researchers guaranteed treating the interviews confidentially and anonymize personal data of the respondents. Further, respondents were informed that they could withdraw from the study at any time, without having to give a reason and without consequences for treatment or access to health care. After reading the information letter, all participants signed an informed consent letter in which they agreed with audiotaping the interviews, analysis of the interviews and use of the interviews in scientific papers.

Author DTH verbally transcribed and translated all interviews into English. We chronologically numbered audio-recording and interview transcripts and stored them on an encrypted server. We did not save any identifiable information, such as names, on any of the files. We did not register participants’ gender or age because we were uncertain whether this was useful for analysis. Table 1 provides a detailed overview of qualitative data collection.

**Table 1:** Qualitative research methods

Source of data	**TB-ENDPoint interviews with Eritrean asylum seekers living in asylum seeker centres in the Netherlands** [11] **TB-ENDPoint interviews with Eritrean refugees living in communities with a maximum duration of stay in the Netherlands of 10 years** [10]
Informed consent	Written a-priori informed consent
Communication	Tigrinya (written and verbal)
Transcript	Verbatim translated from Tigrinya in English (by author DTH^a^)
**Group interviews with Eritreans screened for LTBI (total of 26)**	
Type of interview	Semi-structured group interview
Source of data	Eritrean asylum seekers: *n* = 21, between 2 and 12 participants per interview Eritrean refugees: *n* = 5, between 4 and 6 participants per interview
Participant selection	Participants were invited to participate in group interviews after they had been educated and screened for LTBI
Timing of interview	Directly after LTBI education session and screening
Location	On site of the LTBI screening in a separate room to ensure privacy
	- at one of the asylum seekers centres
	- at one of the Public Health Services
Duration of interviews	Between 30 and 60 min
Incentive	None (drinks and snacks were provided during the interview)
**Individual interviews with Eritreans on LTBI treatment (total of 31)**	
Type of interview	Semi structured individual interviews
Source of data	Eritrean asylum seekers: n = 21
	Eritrean refugees: *n* = 10
Participant selection	TB nurses asked Eritrean participants on LTBI treatment for their consent to be approached by phone by author DTH for an invitation to participate in an individual interview and to set an appointment if willing to participate.
Time	Between 15 and 60 min
Location	Location to the client’s convenience
Incentive	10-euro voucher

*LTBI* Latent tuberculosis infection, *TB* Tuberculosis

^a^All communication, including interviews, with participants were in Tigrinya conducted by the study’s trained research assistant (author DTH), born in Eritrea, with similar background characteristics as the participants, holding a bachelor’s degree in Anthropology

### Analysis

After familiarization with the transcripts, we used an applied thematic approach [25] to analyse our data, which allows for identification of codes and themes in an inductive manner. We identified themes and subthemes from which we developed a coding scheme to guide the coding of transcripts, which we refined along the coding process (by author IS). In regular meetings authors IS, DTH and JS discussed coding, (sub)themes and interpretation of the data [23]. We used MAXQDA (Version 11, VERBI GmbH, Berlin, Germany) to assist in qualitative data analyses.

## Results

We identified five themes: 1) Knowledge and beliefs of participants about TB and LTBI, 2) Attitudes of participants towards TB and LTBI, 3) Felt stigma of participants on LTBI treatment, 4) Enacted stigma of persons on LTBI treatment, 5) Anticipated stigma of participants.

### Knowledge and beliefs about TB and LTBI

#### Background knowledge of TB disease

Most participants had basic knowledge of TB. Some participants, often age 30 or older, explained that they gained initial knowledge about TB through campaigns organized by the local governments in the nineties/early 2000s in Eritrea. They described TB as a serious disease called “the Big Cough”, representing its most common symptom: prolonged persistent cough. Furthermore, most participants said TB could be fatal without treatment.

#### TB transmission and prevention

Despite the education provided in the TB-ENDPoint project, misconceptions about TB transmission and prevention persisted. Both appeared to be embedded in old cultural beliefs about disease transmission and prevention, perceived to be true for diseases in general. Participants described non-relevant TB transmission routes: sharing drinking cups, sharing cigarettes, eating together, cutting with sharp materials and hereditary (parent to child transmission) contaminated water, cold weather, lack of personal hygiene, and mosquito bites. Furthermore, they described the following corresponding prevention measures: use of own drinking cup and eating utensils, get a separate sleeping place, and ventilation of the house (the latter two relevant for contagious TB).

#### Understanding LTBI

Participants shared that they had no knowledge on LTBI prior to the TB-ENDPoint education session. Following the education, most participants described LTBI as a condition without symptoms, in which the sleeping, or hidden, bacteria can cause TB disease later in life. However, some described LTBI as an early stage of TB disease, which was characterized by the absence of symptoms. A few participants described LTBI as “closed” and TB as “open” disease.

#### LTBI and TB versus HIV and AIDS

To differentiate LTBI from TB, many participants compared it to the difference between HIV and AIDS: a person with LTBI or HIV is infected with the bacteria, whereas a person with TB or AIDS is sick. Additionally, some other participants compared the symptoms of TB to those of HIV/AIDS: in both situations the patient would lose weight and would become very weak. Also, when asked to explain the appearance of stigma in the Eritrean society, participants used examples about HIV/AIDS.

*Participant after LTBI screening: “Yes there is of course some fear of isolation. For example, if I have HIV, I would be isolated from the society. There could be people who would try to support you but there are also people who don’t want to come close to you. (…) When one wise person tells you, the disease is nothing serious and you can get treated, then you will get some hope. You can focus on the fight for your health. Otherwise, if I am sick with AIDS and if you and other people isolate me then it means I have no support. In that case you can even get ready to end your life. If I have a disease, I don't want others to know that I am sick, because they are going to isolate me. (…) This is also common for TB disease” [Group interview (GI) 21]*

### Attitudes towards TB and LTBI

Almost all participants said stigma against TB patients occurs within the Eritrean community. Some said that isolating a TB patient would be wrong and they should rather provide support. Most, however, would likely avoid a person with TB disease because of fear of getting infected. Some explained that isolation was not to intentionally hurt or stigmatize the person, but rather to take care and protect oneself.

*Participant after LTBI screening: “In Eritrea if you hear stories that someone has TB, you would be scared to meet him.” [GI 24]*

Participants linked isolation in different ways to knowledge about TB and LTBI. Some said that isolation emerged because of insufficient knowledge about TB and LTBI, and that the projects’ education prior to the LTBI screening had led to understanding and support from community members. On the other hand, few participants said that health education created more awareness and consciousness about severity of TB disease among people who previously had limited knowledge and were more indifferent. This in combination with persistent misconceptions about TB transmission, could lead to other attitudes.

*Participant on LTBI treatment: “A friend of mine in Eritrea had TB, and I used to go with him everywhere. (…) We were together most of the time, he would cough around me, but I never cared much. Maybe because I didn’t know much about the disease. (…) I mean, I did not know how someone can get the disease. Now [after receiving education in the Netherlands] I understand much better.” [Individual interview (II) 29]*

### Felt stigma of persons on LTBI treatment

None of the participants on LTBI treatment described feelings of shame or guilt regarding their LTBI diagnosis. Some persons experienced felt stigma after receiving a letter stating they had a positive test result. They described feelings of shock and disbelief when they found out they tested positive for LTBI. However, the felt stigma was taken away by the extensive explanation about LTBI and treatment possibilities by the TB physician. Participants felt calm and said they were happy “*it was only the TB bacteria and not TB disease”*. Some participants wondered where they could have been infected with TB.

*Participant on LTBI treatment: “I remember something in the military. There was a friend, he was a good barber. He was very thin, and he would keep coughing on top of my head while he was cutting my hair. (…) I always think of TB if people cough around me, no other disease scares me than TB. (…) So, for me, I think it was that friend who transmitted it to me. I am happy that it is not TB disease. (…) Some people decided to stop going to him because they were scared…” [II 24]*

### Enacted stigma of persons on LTBI treatment

Participants who intentionally or unintentionally disclosed their LTBI treatment to relatives and friends received different reactions. Some said they received good support because their friends also participated in the TB ENDPoint education session, which contributed to treatment adherence and completion. Some participants received demotivating reactions: a few community-members told stories about poisonous medication given in the Netherlands. Some participants with LTBI experienced isolation and stigmatization. For example, one participant on LTBI treatment experienced gossip by other Eritreans in the asylum seeker centre.

*Participant on LTBI treatment: “There is two of us taking the treatment and all the people have been whispering and talking about us. They isolate you. I don't tell them the details of what I am doing, but they know that I have the infection. (…) They make you feel as if you bought the disease from the market. It feels as if you have HIV.” [II 31]*

Other participants on TB and LTBI treatment, living in a communal house, experienced similar stigmatization. They explained that, despite the education about TB and LTBI their roommates avoided socializing with them, covered their mouth and nose when interacting, and stressed them to use separate drinking cups and a different toilet. The participants felt hurt, and some isolated themselves. Some participants had addressed the issue with the TB nurse (Ethiopian origin), who organized an additional education session at the communal house they were living in. However, cultural beliefs and fear for disease transmission persisted over new information from TB professionals and the stigmatization continued. Some wished they never were screened. Despite their experience with stigma, participants showed strength and commitment to complete their treatment.

*Participant on LTBI treatment: “When the TB nurse came to give us the education, she told us all that our case cannot be transmitted. But they wouldn't believe that, they still think that it can be transmitted.” [II 22]*

*Participant on TB treatment: ‘Before, we used to share [spoons], and I would give her a bite she would do the same. We were like sisters but that time she said: “I will not use your spoon because you have TB”. I felt really bad. (…) The TB nurse told me that I cannot infect other people, even if I tell that they [roommates] don’t believe it. They would say: “What else do you think they would tell you, maybe they don't want you to be stressed”.’ [II23]*

*Participant on LTBI treatment: “Despite their reaction, I wouldn't want to stop my treatment. It might affect my feelings when they say things in front of me for the moment, but I don't think of stopping my treatment. I am hoping to move to my own house, just to be away from their presence. But for now, until I finish my medicine, I just have to sit in my room.” [II 22]*

### Anticipated stigma

In most interviews, participants said that they would not mind talking about being screened for LTBI. However, out of fear for gossiping and isolation by the community, one would rather conceal a positive test result (see quote GI 21 above). Indeed, some participants on LTBI treatment explained that because of this fear they concealed their LTBI treatment as it would be too difficult to explain the difference between the treatment for infection and disease to others, fearing gossip and isolation.

*Participant on LTBI treatment: “Most of the time people ask you why you are taking the medicine and I cannot say it is for TB because they wouldn't understand the difference. There are also some people if you tell them that you are taking medicine for TB, they wouldn't want to come close to you.” [II 19]*

Some participants said it was nearly impossible to hide their treatment from friends. For example, during social events they had to explain to friends that they could not drink alcoholic beverages because it was not allowed in combination with the medication.

*Participant on LTBI treatment: “When some friends offer you a drink and you would say no, they keep asking why and sometimes they get shocked if you tell them that you are taking medicine for TB. Then you would try to explain to them, but they wouldn't understand it. So, it is always difficult to explain to people. When you try to explain what happened, some would say: "Oh you are becoming like a mouse for an experiment of some medicine”.” [II 18]*

Most participants wished for a change in the Eritrean community. They wished people would start to focus on one’s own health, rather than what others say. Some participants used the following expression: “One who hides his wounds, hides his remedy”, indicating that not going for TB testing because of fear of gossip and isolation could only harm oneself as one will not find out about being infected with TB and therefore will not receive treatment.

*Participant: “I think you should just think about yourself, for example, if I come to do the test and worry about what others may say about me, then it is hiding your own wound.” (…)* [GI 23]

## Discussion

We used data from semi-structured interviews with Eritrean asylum seekers and refugees screened and/or treated for LTBI to gain insights in knowledge, attitudes, beliefs, and stigma related to TB and LTBI. Despite the education session, which all study participants received as part of the LTBI screening, felt and enacted stigma among Eritreans on LTBI treatment emerged because of fear of infection and persisting misconceptions about transmission of TB and LTBI. Our study results show that enacted and anticipated stigma related to LTBI occurred, such as (fear of) isolation by community-members and concealment of LTBI treatment, because of the inability of persons to differentiate LTBI from TB. Consequently, people feared getting infected with TB, which is the most common cause of TB stigma [26–29]. Our study showed that LTBI related stigma is, to a great extent, comparable to TB related stigma. One study among TB patients in Eritrea showed that restrained contacts because of fear of getting infected with TB, gossiping and finger pointing in the community caused enacted and anticipated stigma among TB patients [26]. The comparability of LTBI and TB-related stigma is in line with a previous study among Chinese immigrants in Canada, which linked LTBI related stigma to limited knowledge about LTBI and confusion with TB disease [17]. Although interviewees experienced felt and enacted stigma, which could impede treatment start and completion [29, 30], their strong motivation to prevent becoming sick with TB and complete their treatment resulted in high uptake and completion rates [11, 31].

TB and LTBI education provided throughout the cascade of care improved knowledge, however, did not prevent stigma. This emphasizes that culturally embedded beliefs and fears surrounding TB, which have been present and reinforced throughout a person’s life cannot be reshaped by one single education session [32, 33]. Successful interventions aimed at reducing stigma should include multiple strategies such as counselling, education, and contact, be multi-targeted and oriented, and combine both interpersonal and community levels [32]. Furthermore, education should not only focus on medical aspects of the disease, but also on the psycho-social and economic [34]. Educators -such as TB care staff and community-workers- should thereby appropriately portray messages. Emphasizing the contagiousness of TB rather than effective prevention measures may fail to subduct stigmatizing behaviours and could create and enhance fear [27, 35]. To successfully implement TB prevention programs and to overcome persisting stigmatizing community norms about TB, evidence-based interventions focused on knowledge-shaping and attitude changing are needed [29, 34, 36].

Although TB treatment support interventions reducing stigma have been described [28–30, 36], there is a lack of evidence on effective interventions for persons on LTBI treatment. Moreover, if LTBI treatment support is in place at all, very few countries provide education and psycho-emotional support to those on LTBI treatment [37]. There is thus a need for an overview of evidence-based interventions effectively addressing stigma in LTBI screening and treatment programs, which can be adopted in policies and practice.

We interviewed Eritrean asylum seekers and refugees, a relatively unknown group of interest for TB prevention in the Netherlands, who were screened for LTBI. However, we were unable to interview Eritreans who refused the LTBI screening or treatment. Our results suggest that non-participation might be partially stigma related. It would therefore be important to study reasons for non-participation in LTBI screening and refusal of LTBI treatment. Furthermore, participants may have withheld relevant information, as topics such as TB and stigma are sensitive. Still, this study was able to induce discussion among interviewees and generate valuable information about the TB and LTBI knowledge, attitudes, beliefs, and stigma among Eritreans tested and/or treated for LTBI because the interviews were carried out by an Eritrean interviewer who was able to create a safe and trustful environment. In our study, we invited and interviewed LTBI screening and treatment participants irrespective of age and gender. However, we did not register age and gender of interview participants. While these may play a role in the construction and impact of stigma [13], we did not see this in our data. While we did not register age and gender, when analysing the fragments, we remembered the gender and roughly the age of the participants and could relate gender or age to the fragments. Further research could explore potential gender and age differences in LTBI related stigma and its impact.

## Conclusion

Both anticipated and enacted stigma were reported by Eritrean asylum seekers and refugees who were screened or treated for LTBI. Due to the difficulty to differentiate LTBI from TB and the persistent fear of TB infection and disease, embedded in beliefs, LTBI related stigma was comparable to TB related stigma. Despite a tailored TB and LTBI education program, stigma continuously emerges. Future TB prevention programs should include culturally appropriate education and interventions to address stigma throughout the care cascade. Therefore, there is a need for evidence-based interventions to address TB and LTBI stigma efficiently and effectively in those programs.

## Supplementary information

**Additional file 1.** Description of TB ENDPoint and high-TB-risk migrants.

**Additional file 2.** Overview of the TB and LTBI education session used in the ENDPoint project.

**Additional file 3.** Interview topic guides for group and individual interviews with Eritrean asylum seekers (chapter 1) and refugees (chapter 2).

## Data Availability

This study’s data are not publicly available due to the sensitivity of the data and the privacy of our participants, but are available from the corresponding author (ineke.spruijt@kncvtbc.org) upon reasonable request.

## Abbreviations

LTBI: latent tuberculosis infection
TB: tuberculosis
