# Supplementary material for: Knowledge, attitudes, beliefs, and stigma related to latent tuberculosis infection: a qualitative study among Eritreans in the Netherlands
Source: BMC Public Health. 2020 Oct 23;20:1602. doi: 10.1186/s12889-020-09697-z (PMC7585221; doi:10.1186/s12889-020-09697-z)
Supplement: Supplementary file 1 — Additional file 1. Description of TB ENDPoint and high-TB-risk migrants. [file 12889_2020_9697_MOESM1_ESM.pdf]

Additional file 1

Description of the TB ENDPoint project and definitions of high-TB risk migrant groups

TB ENDPoint project description

The TB ENDPoint project (2016-2020) studied the implementation of an optimized LTBI screening and treatment program among high-TB risk migrants in the Netherlands. The project consisted of three mixed method implementation studies, and a cost effectiveness and impact analysis of the LTBI screening and treatment program.

The first implementation study (1) focused on the implementation of the program as entry-screening among immigrants of all ages from countries with a TB incidence >50/100,000 population, with an intended stay in the Netherlands of six months to ensure ability to complete LTBI screening and, if indicated, treatment.

Definition of immigrant: from the perspective of the country of arrival, a person who moves into a country other than that of his or her nationality or usual residence, so that the country of destination effectively becomes his or her new country of usual residence(2), and not applying for asylum. [project addition to the IOM definition]

The second implementation study (3) focused on the implementation of the program as a follow-up-screening (in addition to mandatory TB entry screening by chest X-ray) among asylum seekers 12 years and older, from countries with a TB incidence of >200/100,000 population (including Eritreans and Ethiopians) living in regular asylum seeker centers and awaiting the decision on their asylum application

Definition of asylum seeker: individuals who are seeking international protection and whose claim has not yet been finally decided on by the country in which he or she has submitted it.(2)

The third implementation study (4) focused on the implementation of the program as a community approach among Eritrean refugees living in Dutch communities for a maximum of ten years. A maximum duration of ten years was chosen as that subgroup of Eritrean refugees was assumed to have the highest risk for TB development.

Definition of refugee: asylum seekers who have been recognized as refugees and granted asylum in the Netherlands within the meaning of the 1951 Convention: a refugee is a person who, owing to a well-founded fear of persecution for reasons of race, religion, nationality, membership of a particular social group or political opinion, is outside the country of his nationality and is unable or, owing to such fear, is unwilling to avail himself of the protection of that country; or who, not having a nationality and being outside the country of his former habitual residence as a result of such events, is unable or, owing to such fear, is unwilling to return to it.(2)

LTBI screening and treatment program

In the Netherlands, TB care and prevention activities are done by specialized TB healthcare workers of local public health services. The LTBI screening is conducted by TB medical technical assistants and consists of a health questionnaire and LTBI testing. The health questionnaire assesses prevalent symptoms indicative of TB, TB history, comorbidities, medication use, BCG vaccination status and HIV status. If clients have complaints indicative of TB disease, a chest X-ray is taken, and the client will receive a consultation with the TB physician. LTBI testing is done either by TST&IGRA (TST induration >5mm, followed by confirmatory IGRA) or IGRA alone. LTBI treatment consist of three-month daily Isoniazid and Rifampicin combination therapy and is supervised by TB nurses.

References

1. Spruijt I, Erkens C, Suurmond J, Huisman E, Koenders M, Kouw P, et al. Implementation of latent tuberculosis infection screening and treatment among newly arriving immigrants in the Netherlands: A mixed methods pilot evaluation. PLoS One. 2019;14(7):e0219252.
2. Internation Organization for Migration (IOM). Key Migration Terms Grand-Saconnex, Geneva: IOM; 2020 [Available from: https://www.iom.int/key-migration-terms.
3. Spruijt I, Tesfay Haile D, Suurmond J, van den Hof S, Koenders M, Kouw P, et al. Latent tuberculosis screening and treatment among asylum seekers: a mixed methods study. Eur Respir J. 2019.
4. Spruijt I, Haile DT, Erkens C, van den Hof S, Goosen S, Ten Kate A, et al. Strategies to reach and motivate migrant communities at high risk for TB to participate in a latent tuberculosis infection screening program: a community-engaged, mixed methods study among Eritreans. BMC Public Health. 2020;20(1):315.
