# Supplementary material for: Knowledge, attitudes, beliefs, and stigma related to latent tuberculosis infection: a qualitative study among Eritreans in the Netherlands
Source: BMC Public Health. 2020 Oct 23;20:1602. doi: 10.1186/s12889-020-09697-z (PMC7585221; doi:10.1186/s12889-020-09697-z)
Supplement: Supplementary file 2 — Additional file 2. Overview of the TB and LTBI education session used in the ENDPoint project. [file 12889_2020_9697_MOESM2_ESM.pdf]

Additional file 2

Overview of the TB and LTBI education session used in the ENDPoint project

Subject 1 Tuberculosis

ቀዳማይ ኣርእስቲ ሕማም ዓባይ ሰዓል

The knowledge of the target group about tuberculosis is expected to be low. It is therefore, important to start increasing the basic knowledge about tuberculosis of the target population. Without this basic knowledge, it is hard to explain latent tuberculosis infection and screening. The basic information about why the tuberculosis screening is organized, what it entails, who is screened, etcetera, is not well comprehended by the target population. Therefore, it is important to explain the policy for tuberculosis screening in the Netherlands.

SLIDE 2: What is tuberculosis?
Tuberculosis is an infectious disease caused by bacteria.

SLIDE 2: ዓባይ ሰዓል ማለት እንታይ ማለት'ዩ?
ዓባይ ሰዓል ለበዳዊ ሕማም ኮይኑ ብማይኮ-ባክተሪዩም ቱበርኩሎሲስ ዝበሃል ባክተርያ ይመጽእ።

SLIDE 3: Where in the body can tuberculosis occur? Tuberculosis can affect the lungs, but it can also affect other parts of the body, such as lymph nodes, the brain, the eyes, the kidneys, or the spine.

SLIDE 3: ዓባይ ሰዓል ኣበይ ክፍሊ ኣካላትና ክንሓምም ንኽእል?

ሕማም ዓባይ ሰዓል ብቐንዱ ሳንቡእ ዘጥቅዕ ኮይኑ፡ ካልእ ክፍሊ ኣካላትና'ውን ከጥቅዕ ይኽእል። ንኣብነት ኣብ ጽኪታት፡ ስርዓተ ኣእምሮ፡ ዓይኒ፡ ኮላሊትን ዓንድ-ሕቖን.።

SLIDE 4: What are symptoms of tuberculosis?
Tuberculosis can cause symptoms like coughing (more than 2 weeks), fever, weight loss or poor appetite, night sweats. Some people with lung tuberculosis cough up blood.

SLIDE 4: ምልክታት ሕማም ዓባይ ሰዓል እንታይ እዮም?
ሕማም ዓባይ ሰዓል ነዞም ዝስዕቡ ምልክታት ከርኢ ይኽእል፡ ንልዕሊ 2 ሰሙን ዝጸንሕ ሰዓል፡ ረስኒ፡ ምጉዳል ሚዛን ሰብነት፡ ሸውሃት ምዕጻው፡ ናይ ለይቲ ረሃጽ። ገለ ናይ ስንቡእ ሕማም ሰዓል ዘለዎም ሰባት ደም ዝተሓወሶ ዓኽታ ክረኣዮም ይኽእል።

SLIDE 5: How can you get tuberculosis?
Tuberculosis is spread from person to person through the air. You can get tuberculosis when a person with tuberculosis in the lungs sneezes or coughs. The tuberculosis bacteria can then get into the air in little droplets. Another person can inhale the air with the tuberculosis bacteria, he or she may then become infected.

SLIDE 5: ሕማም ዓባይ ሰዓል ብኸመይ ክለኸፈካ ይኽእል?
ሕማም ዓባይ ሰዓል ካብ ሰብ ናብ ሰብ ብንፋስ ወይ ምስትንፋስ ይመሓላለፍ። ሓደ ናይ ሳንቡእ ዓባይ ሰዓል ዘለዎ ሰብ ምስ ዘሀንጥስ ወይ ዝስዕል ክለኽፈካ ይኽእል። እቲ ናይ ዓባይ ሰዓል ባክተርያ ካብ ውሽጢ ስርዓተ ትንፋስ ምስዝወጹ ደቐቕቲ ማያዊ-ነጠብጣብ ናብ ኣየር ይሕወስ። ዝኾነ ሰብ ነቲ ዝተበከለ ኣየር ምስዘተንፍስ፡ ባክተርያ ዓባይ ሰዓል ናብ ውሽጡ ይኣትዉ በዚ ድማ ክሓምም ይኽእል።

SLIDE 6: Who can get tuberculosis?

Everyone can get infected with the tuberculosis bacteria. That is why everybody can get tuberculosis disease. Even the king and queen can get tuberculosis disease.

There are some people who have a higher risk that the tuberculosis bacteria is in the body. This is because they have had a higher chance of being exposed to the bacteria. For example: persons who have been in contact with TB patients, health care workers, persons who live / lived in countries where tuberculosis is still highly prevalent.

SLIDE 6: ሕማም ዓባይ ሰዓል ክሓምም ዝኽእል መን'ዩ?

ዝኾነ ሰብ ብዓባይ ሰዓል ክልከፍ ይኽእል'ዩ፡ ስለዝኾነ ድማ ዝኾነ ሰብ ሕማም ዓባይ ሰዓል ናይ ምሕማም ተኽእሎ ኣለዎ። ዋላ'ውን ንጉስን ንግስትን ናይ ሃገረ ኔዘርላንድ ክሓሙ ይኽእሉ።

እንተኾነ ገለ ሰባት ዓባይ ሰዓል ናይ ምልካፍ ዝለዓለ ተኽእሎ ኣለዎም፡ እዚ ማለት ብባክተርያ ዓባይ ሰዓል ክልከፍሉ ዝኽእሉ ዝሰፍሐ ዕድላት ወይ ኣጋጣሚታት ኣለዎም ወይ ኔርዎም ማለት እዩ። ንኣብነት፡ ምስ ሓማም ዓባይ ሰዓል ዘለዎ ሰብ ብኣካል ዝነበሩ፡ ሰራሕተኛታት ማእከላት ጥዕና፡ ኣብተን ዓባይ ሰዓል ብብዝሒ ዝረኣየለን ሃገራት ዝነበሩ/ዝመጹ።

SLIDE 7: Quiz

SLIDE 7: ሕቶ

SLIDE 8: Tuberculosis in the world

Tuberculosis is still highly prevalent in the world. Tuberculosis is the number one killer infectious disease in the world. Even more than HIV or malaria. Every year 10.4 million people develop tuberculosis disease worldwide. Every year, 1.7 million people die of tuberculosis disease.

SLIDE 8: ሕማም ዓባይ ሰዓል ብዓለም ደረጃ ከመይ ይመስል!

ሕማም ዓባይ ሰዓል ብዓለም ደረጃ ገና ግኑን ሕማም'ዩ። ካብ ኩሎም ተማሓላለፍቲ ሕማምት፡ ብዓለም ደረጃ ቀዳማይ ቀታሊ ሕማም ኮይኑ ተመዝጊቡ ይርከብ። እዚ ማለት ልዕሊ ኤይድስን ሕማም ዓሶን ማለት'ዩ። ብዓለም ደረጃ ዓመታዊ 10.4 ሚልዮን ህዝቢ ሕማም ዓባይ ሰዓል ይሓምም። ከምኡ'ውን ዓመታዊ 1.7 ሚልዮን ህዝቢ ብሰንኪ ሕማም ዓባይ ሰዓል ይመውት።

SLIDE 9: Tuberculosis in the Netherlands

Tuberculosis was a very common disease in the Netherlands in the past. In the past, there were no medication for tuberculosis. In the Netherlands, there were special hospitals for tuberculosis patients. Their treatment consisted of fresh outdoor air and bedrest. Some people were in the hospital for more than a year. A lot of people died from tuberculosis disease back then. Luckily, we have good medication since the sixties. Because of these medication, improved good hygiene, social welfare and a very good tuberculosis control and care, tuberculosis is now not so prevalent anymore in the Netherlands.

SLIDE 9: ሕማም ዓባይ ሰዓል ኣብ ሃገረ ኔዘርላንድ

ኣብ ሃገረ ኔዘርላንድ ቅድሚ ነዊሕ ዓመታት ሕማም ዓባይ ሰዓል ኣዝዩ ግኑን ሕማም'ዩ ኔሩ። ኣብቲ እዋን መድሃኒት'ውን ኣይነበሮን። ኣብቲ እዋን ኣብ ሃገረ ኔዘርላንድ ንሕሙማት ዓባይ ሰዓል ዝኸውን ፍሉይ ሆስፒታላት ኔሩ። እቲ ናይ ፍወሳ መስርሕ ድማ፡ ጽሩይ ኣየር ብምሃብን ኣብ ዓራት ብምድቃስን ጥራይ'ዩ ኔሩ። ገለ ሰባት ኣብቲ ሆስፒታላት ንልዕሊ ዓመት ይድቅሱ ኔሮም። ኣብቲ እዋን ብዙሓት ሰባት ብሕማም ዓባይ ሰዓል ይሞቱ ኔሮም። ካብ ከባቢ 1960'ታት ጀሚሩ ግና መድሃኒት ናይቲ ሕማም ተረኺቡ። በዚ መሰረት ነቲ ሕማም ዝፍውስ መድሃኒት፡ ዝተመሓየሸ ጽሬት ጥዕና፡ ማሕበራዊ ድሕነት፡ ዝማዕበለ ኣተኣላልያ ሕማም ዓባይ ሰዓል ብምኽታል፡ ኣብዚ እዋን ሕማም ዓባይ ሰዓል ኣብ ኔዘርላንድ ኣብ ኣዝዩ ዝተሓተ ደረጃ ይርከብ።

SLIDE 10: Tuberculosis screening in the Netherlands

In the Netherlands, all migrants from countries were tuberculosis is still prevalent must be screened for tuberculosis at entry. For this screening, a radiological photo of the lungs is taken. On this photo, the tuberculosis doctor can see if someone has tuberculosis disease of the lungs.

All migrants who come from countries were tuberculosis is still highly prevalent are offered a voluntarily radiological screening for tuberculosis. Every 6 months for a period of 2 years, that person will receive an invitation to get a photo of the lungs.

The screening for tuberculosis is conducted because we want everybody who has tuberculosis in the Netherlands to receive treatment with medication and good care. In the Netherlands, we do not want anyone to die from tuberculosis because we can provide care. A second reason for the screening is we want to make sure that someone with lung tuberculosis does not spread the disease to other persons, so we can prevent other persons from getting tuberculosis disease.

SLIDE 10: መርመራ ሕማም ዓባይ ሰዓል ኣብ ሃገረ ኔዘርላንድ

ኣብ ሃገረ ኔዘርላንድ፡ ኩሎም ስደተኛታት ካብተን ሕማም ዓባይ ሰዓል ዝኽሰተለን ሃገራት ዝመጹ ኣብዚ ሃገር ክኣትዉ ከለዎ ናይ ግድን መርመራ ዓባይ ሰዓል ክገብሩ ይግደዱ። ኣብቲ ዝግበር መርመራ ናይ ሳንቡእ ስእሊ ወይ X-ray ይውሰደሎም። ኣብቲ ስእሊ ምርኩስ ብምግባር ድማ፡ ናይ ዓባይ ሰዓል ሓኪም እቲ ሰብ ናይ ሳንቡእ ዓብይ ሰዓል ኣለዎን የብሉን የረጋግጽ።

ኩሎም ካብተን ሕማም ዓባይ **ሰዓል ብዝለዓለ ዝረኣየለን** ሃገራት ዝመጹ ስደተኛታት ወለንታዊ ናይ ሳንብእ ስእሊ / x-ray መርመራ ክገብሩ ዕድል ይወሃቦም። በዚ መሰረት ንክልተ ዓመት ዝቕጽል መርመራ ናይ ሳንቡእ ስእሊ / X-ray ንምግባር ኣብ ሸሽዱሽተ ወርሒ ዕድመ ይግበረሎም።

መርመራ ሕማም ዓባይ ሰዓል ነካይደሉ ቀንዲ ምኽንያት፡ ኣብ ሃገረ ኔዘርላንድ ዝኾነ ሕማም ዓባይ ሰዓል ዘለዎ ሰብ ግቡእ መድሃኒት ብምውሳድን ግቡእ ክንክን ብምርካብን ክሓዊ ስለንደልዮ'ዩ። ኣብ ሃገረ ኔዘርላንድ ዋላ ሓደ ሰብ'ውን ይኹን ብሕማም ዓባይ ሰዓል ክመውት ኣይንደልን ኢና፡ ምኽንያቱ ክንሕክሞ ዓቕሚ ስለዘለና። መርመራ ሕማም ዓባይ ሰዓል ንገብረሉ ካልኣይ ምኽንያት ድማ፡ ሓደ ናይ ሳንቡእ ሕማም ዓባይ ሰዓል ዘለዎ ሰብ ነቲ ሕማም ብምስፍሕፋሕ ንኻልኦት ከይልክም ንምክልኻል ኮይኑ፡ በዚ መሰረት ካልኦት ሰባት ሕማም ዓባይ ሰዓል ካብ ምሕማም ነድሕን ማለት'ዩ።

SLIDE 11: What happens if the doctor finds tuberculosis through the screening?

Everyone who has tuberculosis disease in the Netherlands will be offered treatment with medication by the doctor. Beside the medication, the patient will receive support from the tuberculosis nurse. In the Netherlands, everybody has the right for tuberculosis care: even persons who reside illegal, homeless persons, and drug users.

SLIDE 11: ካብቲ ዝካየድ መርመራ እቲ ሓኪም ሕማም ዓባይ ሰዓል ከምዘሎ ምስዘረጋግጽ፡ እቲ ዝስዕብ መስርሕ እንታይ ይኸውን?

ኣብ ሃገረ ኔዘርላንድ፡ ዝኾነ ሕማም ዓባይ ሰዓል ዘለዎ ሰብ ካብ ሓኪም ብመድሃኒት ዝውሰድ ፍወሳ ክከታተል ዕድል ይወሃቦ። ብዘይካ እቲ ዝውስድ መድሃኒት፡ እቲ ሕሙም ካብ ናይ ዓባይ ሰዓል ነርስ (ኣላይት ሕሙም) ተወሳኺ ሓገዝ ይረክብ። ኣብ ሃገረ ኔዘርላንድ፡ ኩሉ ሰብ ናይ ሕማም ዓባይ ሰዓል ፍወሳ ክወስድ መሰለ ኣለዎ፡ ዋላ'ውን ብዘፍቓድ ዝነብሩ ሰባት፡ ግዳም ሓደራት፡ ግዙኣት ወልፊ ሓሽሽ ወይ ዕጸ ፋርስ ወዘተ።

SLIDE 12: Film: TB: the real story

SLIDE 12: ሕማም ዓባይ ሰዓል፡ ሓቀኛ ዛንታ

Subject 2 ካልኣይ ኣርእስቲ
Latent tuberculosis infection
መልከፍቲ ባክተርያ ዓባይ ሰዓል

There is a need for explanation of the difference between LTBI and active tuberculosis. This information plays a fundamental role in understanding the purpose and objective of the LTBI screening by the target group.

ፍልልይ ኣብ ምንጎ መልከፍቲ ባክተርያ ዓባይ ሰዓልን ንጡፍ ሕማም ዓባይ ሰዓልን ምግላጽ ኣገዳሲ'ዩ። እዚ ሓበሬታ ኣገዳስነትን ዕላማን ናይዚ ዝግበር መርመራ መልከፍቲ ባክተርያ ዓባይ ሰዓል ኣብ ምንጎ'ዚ ክፋል ሕብረተሰብ ንምርዳእ ዓቢ ኣስተዋጽኦ ኣለዎ።

SLIDE 13: Can we prevent someone from getting tuberculosis disease?
Yes, we can.

SLIDE 13: ሓደ ሰብ ሕማም ዓባይ ሰዓል ካብ ምሕማም ከንድሕኖ ንኽእሎ?
እወ ንኽእል!

SLIDE 14: How can tuberculosis be prevented?
A person can get infected with the tuberculosis bacteria when someone in his surroundings with lung tuberculosis coughs. If this person inhales the droplets with the tuberculosis bacteria, three things can happen:

1) the body is strong and can kill the bacteria immediately → you will have no infection
2) the body cannot control the bacteria. The bacteria remain very active and causes damage to the body → the person gets tuberculosis disease
3) the body can control the bacteria. The bacteria are still in the body but it is inactive. We also say the bacteria is sleeping. → The is called latent tuberculosis infection.
   Unfortunately, it is possible that even after 10 years the sleeping tuberculosis bacteria can wake up and become active. It will then cause tuberculosis disease.

We can prevent tuberculosis disease. This is done by testing people for the presence of the tuberculosis bacteria in the body (when it is inactive or sleeping). If the test result tells the doctor that a person has the bacteria in their body, then we can treat that person with medication. The medication will then kill the sleeping bacteria.

SLIDE 14: ሕማም ዓባይ ሰዓል ብኸመይ ክንከላኸሎ ወይ ከነወግዶ ንኽእል?
ሓደ ሰብ ብባክተርያ ዓባይ ሰዓል ክልከፍ ዝኽእል፡ ሓደ ናይ ሳንቡእ ሕማም ዓባይ ሰዓል ዘለዎ ሰብ ኣብ ከባቢኡ ምስዝስዕል'ዩ። እዚ ሰብ ነቲ ብባክተርያ ዓባይ ሰዓል ዝተበከለ ኣየር ንውሽጢ ምስዘተንፍሶ፡ ሰለስተ ነገራት ከጋጥም ይኽእል፡-

1) እቲ ሰብ ናይ ምጽዋር ዓቕሙ ሓያል ይጸንሕ'ሞ ነቲ ባክተርያ ብቐጥታ ይቐትሎ → ስለዚ ዝኾነ መልከፍቲ ኣየጋጥምን ማለት'ዩ
2) እቲ ሰብ ነቲ ባክተርያ ክጻወሮ ኣይክእልን። እቲ ባክተርያ ድማ ንጡፍ ብምኻን ሳዕቤን የኸትለሉ → ስለዚ እቲ ሰብ ሕማም ዓባይ ሰዓል ይሓምም ማለት'ዩ
3) እቲ ሰብ ነቲ ባክተርያ ክጻወሮ ይኽእል። እቲ ባክተርያ ገና ኣብቲ ኣካላት'ቲ ውልቀ-ሰብ ኣሎ ግን ብዘይንጡፍ ኩነታት። እዚ ዝደቀሰ ባክተርያ ኢልና ክንጽውዖ ንኽእል። → ነዚ ድማ መልከፍቲ ባክተርያ ዓባይ ሰዓል ኢልና ንጽውዖ።
   እንተኾነ ግን፡ እዚ ዝደቀሰ ባክተርያ ዋላ'ውን ድሕሪ ዓሰርተ (10) ተበራቢሩ ንጡፍ ክኸውን ይኽእል። በዚ መሰረት ድማ ሕማም ዓባይ ሰዓል ከኸትል ይኽእል።

ሕማም ዓባይ ሰዓል ካብ ምሕማም ክንከላኸከ ንኽእል ኢና። እዚ ነቲ ዝደቀሰ ባክተርያ ዓባይ ሰዓል ንምልላይ፡ መርመራ ብምግባር ዝካየድ ኣዩ። እቲ ሓኪም ውጽኢት መርመራ ብምርኣይ፡ ባክተርያ ዓባይ ሰዓል ኣብ ኣካል'ቲ ሰብ ከምዘሎ እንተደኣ ኣረጋጊጹ፡ ነቲ ሰብ መድሃኒት ብምሃብ ክንፍውሶ ንኽእል። እቲ ማለት እቲ መድሃኒት ነቲ ዝደቀሰ ባክተርያ ይቐትሎ ማለት'ዩ።

SLIDE 15: What is the difference between tuberculosis infection and tuberculosis disease?

The biggest difference between a person with latent tuberculosis infection and tuberculosis disease is that the person with tuberculosis disease will be sick: he or she will have symptoms. A person with latent tuberculosis infection does not have symptoms because the bacteria are sleeping. He or she feels normal. Besides the absence of symptoms, the lung photo of someone with LTBI does not show abnormalities. The photo does often show abnormalities when someone has lung tuberculosis. Someone with latent tuberculosis infection is not infectious: he or she cannot spread the bacteria to other persons. Someone with lung tuberculosis can spread the bacteria to other persons.

SLIDE 15: ኣብ ምንጎ መልከፍቲ ባክተርያ ዓባይ ሰዓልን ሕማም ዓባይ ሰዓልን ዘሎ ፍልልይ እንታይ'ዩ?

እቲ ዝዓበየ ፍልልይ ኣብ መንጎ መልከፍቲ ባክተርያ ዓባይ ሰዓል ዘለዎ ሰብን ሕማም ዓባይ ሰዓል ዝሓመመ ሰብን፡ እቲ ሕማም ዓባይ ሰዓል ዘለዎ ሰብ ሓሚሙ'ዩ ዘሎ፡ ከምኡ'ውን ምልክታት ናይቲ ሕማም ይህልዎ ማለት'ዩ። እቲ መልከፍቲ ባክተርያ ዓባይ ሰዓል ዘልዎ ሰብ ብኣንጻሩ ዝኾነ ምልክታት ናይቲ ሕማም የብሉን ምኽንያቱ እቶም ባክተርያ ድቁሳት ስለዝኾኑ። ዝኾነ ዘይንቡር ነገር'ውን ኣይስምዖን። ብዘይካ ምልክታት ሕማም ዘይምርኣይ፡ ናይ ሳንቡእ ስእሊ / X-ray ናይቲ መልከፍቲ ባክተርያ ዘልዎ ሰብ ዝኾነ ዘይንቡር ምልክት ኣየርእይን'ዩ። ብኣንጻሩ ሓደ ናይ ሳንቡእ ዓባይ ሰዓል ዘለዎ ሰብ ኣብቲ ናይ ሳንቡእ ስእሊ / X-ray ዘይንቡር ምልክታት ክርአ ይኽእል። ሓደ መልከፍቲ ባክተርያ ጥራይ ዘለዎ ሰብ ተላባዒ ኣይኮነን፡ እቲ ባክተርያ ናብ ካልኦት ሰባት ከመሓላልፍ ኣይክእል'ዩ። ሓደ ናይ ሳንቡእ ሕማም ዓባይ ሰዓል ዘለዎ ሰብ ብኣንጻሩ እቲ ባክተርያ ናብ ካልኦት ከመሓላልፍ ይኽእል።

SLIDE 16: Who have highest risk of developing tuberculosis disease?

All persons who have been recently infected (<2.5 years) have higher chance of developing tuberculosis disease than persons who have been infected a long time ago. Some people have a higher chance of developing tuberculosis disease because they have a weak immune system. Because they have a weak immune system the body cannot fight the tuberculosis bacteria. Therefore, the bacteria have a high chance of being able to cause tuberculosis disease. For example: persons with HIV/aids, persons with diabetes or persons with cancer. Some people get medication that weaken the immune system, for example persons who have had an organ transplantation. Also, these persons have higher chance of developing tuberculosis disease.

SLIDE 16: ሕማም ዓባይ ሰዓል ናይ ምምዕባል ዝለዓለ ተኽእሎ ዘለዎም መን'ዮም?

ኩሎም ኣብ ቀረባ እዋን ዝተለኽፉ ሰባት (እቲ መልከፍቲ ኣብ ውሽጢ 2 ዓመትን ፈረቓን ምስዝኸውን)፡ ካብቶም ቅድሚ ነዊሕ ዓመታት ዝተለኽፉ ሰባት እቲ ሕማም ናይ ምምዕባል ዝለዓለ ተኽእሎ ኣለዎም። ብተወሳኺ ናይ ምጽዋር ዓቕሞም ዝተዳኸመ ወይ ትሑት ዝኾነ ሰባት'ውን እቲ ሕማም ናይ ምምዕባል ተኽእሎ ኣለዎም። ናይ ምጽዋር ዓቕሞም ድኹም ወት ትሑት ስለዝኾነ፡ ነቲ ባክተርያ ክቃለስዎ ኣይክእሉን። ስለዚ እቲ ባክተርያ ሕማም ክኸትለሎም ዝለዓለ ተኽእሎ ይህልዎ። ንኣብነት፡ ምስ ሕማማት ኤይድስ ወይ HIV፡ ሕማም ሽኮር፡ መንሽሮ ዝነብሩ ሰባት ክንጠቅስ ንኽእል። ከምኡ'ውን ናይ ምጽዋር ዓቕሞም ከዳኽም ዝኽእል ከቢድ መድሃኒታት ዝወስዱ ሰባት፡ ንኣብነት ፡ መጥባሕቲ ምቕያር ናውቲ ኣካላት ክንጠቅስ ንኽእል። ኩሎም እዞም ዝጠቐስናዮም ሰባት ሕማም ዓባይ ሰዓል ናይ ምምዕባል ዝለዓለ ተኽእሎ ኣለዎም'።

SLIDE 17: Quiz

SLIDE 17: ሕቶ

SLIDE 18: How can we diagnose latent tuberculosis infection?

There are two ways to diagnose latent tuberculosis infection:

1) **Tuberculin Skin Test**
2) **Blood test**

SLIDE 18: መልከፍቲ ባክተርያ ዓባይ ሰዓል ብኸመይ ክንፈልጦ ወይ ክንምርምሮ ንኽእል?

መልከፍቲ ባክተርያ ዓባይ ሰዓል ንምምርማር ክልተ መገድታት ክንጥቀም ንኽእል፡

1) ናይ ቆርበት መርመራ
2) ናይ ደም መርመራ

SLIDE 19: What happens if someone has a positive blood test?

If the result of the blood test is positive, it means that the tuberculosis bacteria are detected in the body. The person will then receive an appointment with the tuberculosis doctor at the GGD. The doctor will do further examination. The doctor will take a photo of the lungs. The doctor will also ask questions. With these examinations, the doctor will exclude tuberculosis disease, and diagnose latent tuberculosis infection.

If the blood test is negative, it means that the person does not have the tuberculosis bacteria in the body at this moment. The person does not need further examination. The person does not have to go to the doctor.

SLIDE 19: ደም መርመራ ናይ ሓደ ሰብ ኣወንታ ወይ ፖሰቲቭ ምስዝኸውን ዝስዕብ መስርሕ እንታይ'ዩ?

እቲ ውጽኢት መርመራ ደም ኣወንታ ወይ ፖሰቲቭ እንተድኣ ኮይኑ፡ ባክተርያ ዓባይ ሰዓል ኣብቲ ሰብ ተረኺቦም ኣለው ማለት'ዩ። በዚ ምኽንያት እቲ ሰብ ምስ ሓኪም ዓባይ ሰዓል ኣብ ኼ ኼ ደ ንምርኻብ ናይ ቆጸራ ዕድመ ይቕበል። እቲ ሓኪም ተወሳኺ መርመራታት የካይድ። እቲ ሓኪም ናይ ሳንቡእ ስእሊ ይወስድ፡ ከምኡ'ውን ሕቶታት ይሓትት። እዚ መርመራታት ብምግምጋም፡ እቲ ሓኪም ሕማም ዓባይ ሰዓል ከምዘይኮነ፡ መልከፍቲ ባክተርያ ዓባይ ሰዓል ምኻኑ የረጋግጽ።

እቲ ውጽኢት መርመራ ደም ኣሉታ ወይ ነጋቲቭ ምስዝኸውን፡ እቲ ሰብ ኣብዚ ግዜ ዝኾነ ባክተርያ ዓባይ ሰዓል ኣብ ኣካላቱ የብሉን ማለት'ዩ። እቲ ሰብ ተወሳኺ መርመራታት ኣይገብርን፡ ናብ ሓኪም ምቕራብ'ውን ኣየድልዮን ማለት'ዩ።

SLIDE 20: Can latent tuberculosis infection be treated?

Yes. Latent tuberculosis infection can be treated with medication / tablets. These must be taken every day at the same moment. The treatment for tuberculosis infection takes 3 months.

SLIDE 20: መልከፍቲ ባክተርያ ዓባይ ሰዓል ክፍወስ ይኽእል'ዶ?

መልከፍቲ ባክተርያ ዓባይ ሰዓል መድሃኒት ወይ ከኒና ክፍወስ ይኽእል። እቲ መድሃኒት መዓልታዊ ኣብ ውሱን ሰዓት ክውሰድ ይግባእ። ፍወሳ መልከፍቲ ባክተርያ ዓባይ ሰዓል ሰለስተ (3) ወርሒ ይወስድ።

SLIDE 21: Quiz

SLIDE 21: ሕቶ

SLIDE 22: Why should you do the test for latent tuberculosis infection?

In the horn of Africa there is still a lot of tuberculosis. Also in Eritrea. In the Netherlands, we see a lot of Eritreans develop tuberculosis after they have arrived in the Netherlands. This happens sometimes even after 5 or more years. You have had a screening for tuberculosis infection in the past. This was an X-ray. However, as you have learned, an x-ray can only detect active lung tuberculosis. By doing the blood test we can see if you have the tuberculosis bacteria in your body. If you have the tuberculosis bacteria in your body, we will offer you

free treatment. The medication will kill the tuberculosis bacteria. In this way we can make sure that you do not develop tuberculosis in the future.

SLIDE 22: ስለምንታይ መርመራ መልከፍቲ ባክተርያ ዓባይ ሰዓል ምግባር የድልየኩም?

ኣብ ቀርኒ ኣፍሪቃ ከምኡ'ውን ኣብ ኤርትራ ሕማም ዓባይ ሰዓል ገና ብብዝሒ ይርአ። ኣብ ሃገረ ኔዘርላንድ ውን'ተኾነ፡ ኤርትራውያን ኣብዚ ሃገር ድሕሪ ምእታዎም ሕማም ዓባይ ሰዓል ብብዝሒ ከማዕብሉ ይረኣዩ። እዚ ሓደ ሓደ እዋን ድሕሪ ሓሙሽተ (5) ዓመትን ልዕሊኡን'ውን ዘጋጥም'ዩ። ቅድሚ ሕጂ ናይ ዓባይ ሰዓል መርመራ ጌርኩም ኔርኩም። እዚ ድማ ናይ ሳንቡእ ስእሊ ወይ X-ray እዩ ኔሩ። እንተኾነ፡ ከምቲ ኣቐዲምና ዝተመሃርናዮ X-ray ንጡፍ ናይ ሳንቡእ ዓባይ ሰዓል ጥራይ'ዩ ክለሊ ወይ ክምርምር ዝኽእል። ደም መርመራ ብምግባር ግን እቲ ባክተርያ ኣብ ኣካላትኩም ኣሎ'ዶ የለን ከነረጋግጽ ንኽእል። እቲ ባክተርያ ኣብ ኣካላትኩም እንተድኣ ተረኺቡ፡ ነጻ ናይ ፍወሳ ዕድል ንህበኩም። እቲ መድሃኒት ነቲ ባክተርያ ዓባይ ሰዓል ክቕትሎ እዩ። በዚ መገዲ፡ ንመጻኢ ሕማም ዓባይ ሰዓል ካብ ምምዕባል ትድሕኑ ማለት'ዩ።

Health education

Latent Tuberculosis Infection : screening and treatment

KNCV
TUBERCULOSISFOUNDATION
To eliminate TB

What is Tuberculosis?

- Infectious disease
- Caused by a bacteria

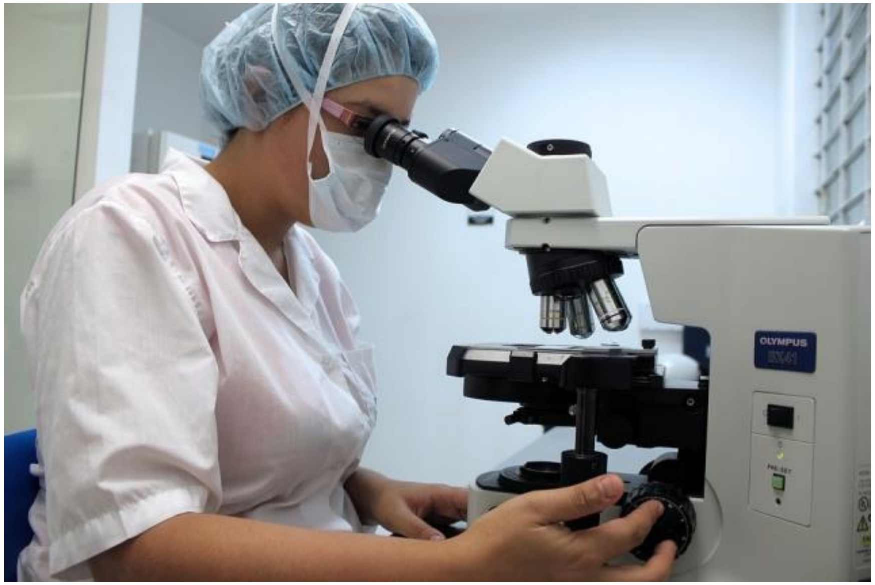

Microscope

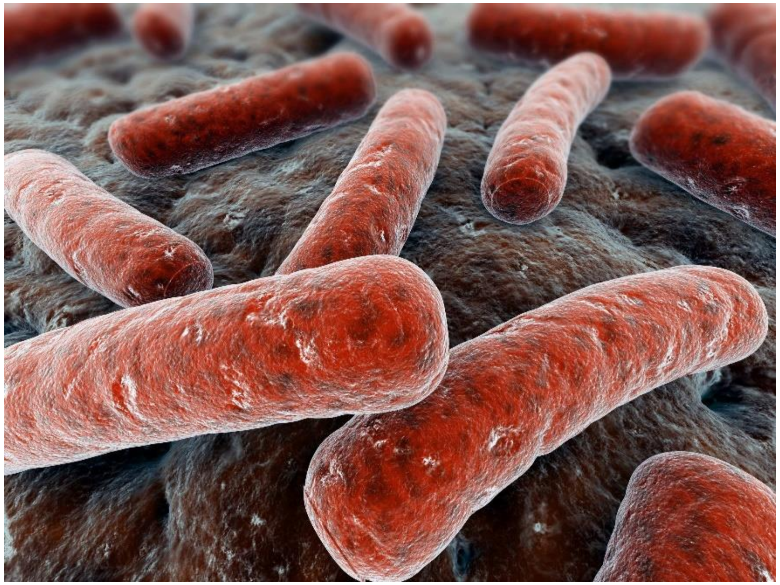

Mycobacterium tuberculosis

Where can you get tuberculosis?

Tuberculosis can affect many parts of the body

Tuberculosis of the lungs

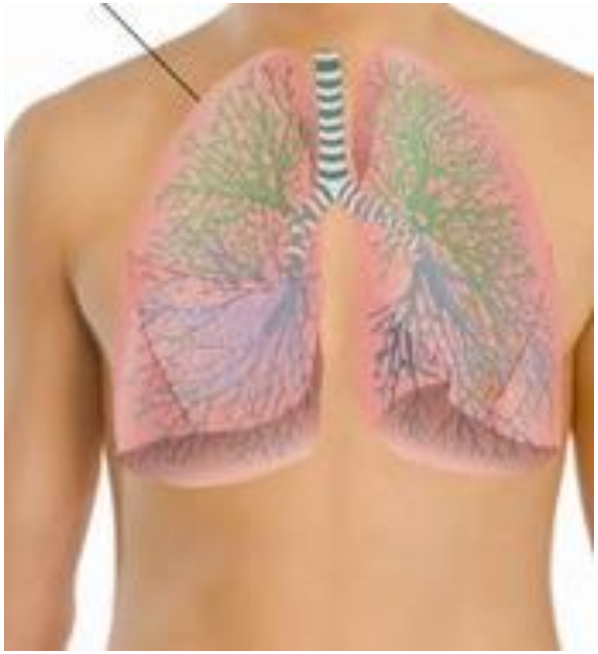

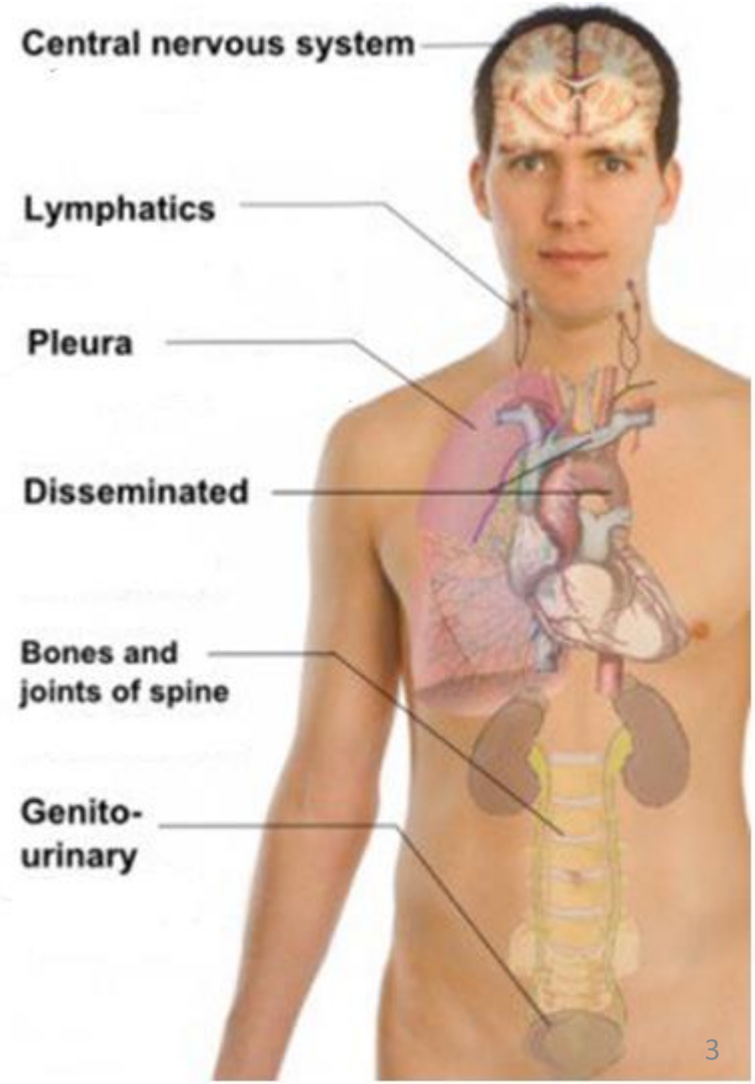

What are the symptoms of tuberculosis disease?

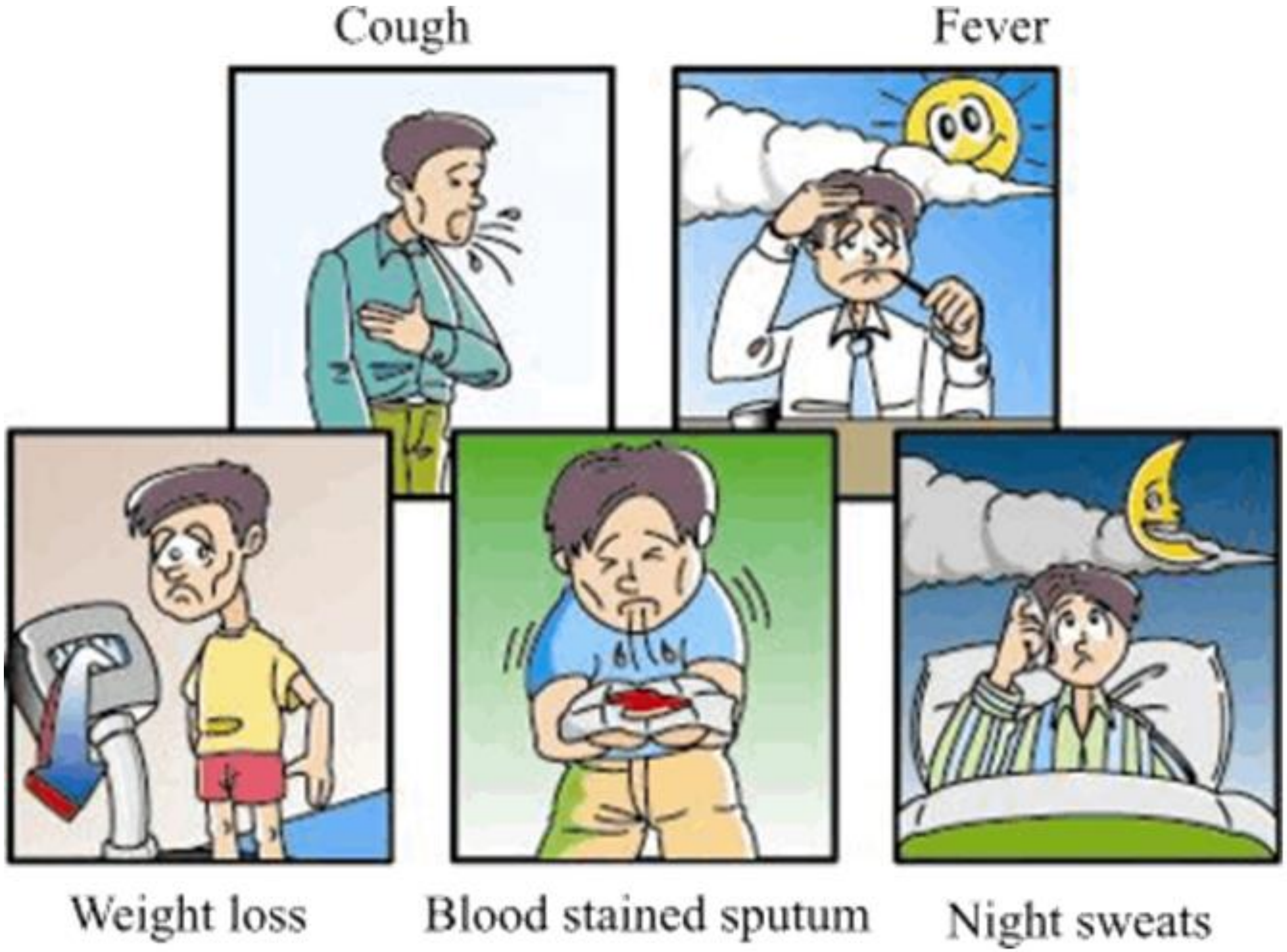

How can you get Tuberculosis?

Tuberculosis is spread through the air

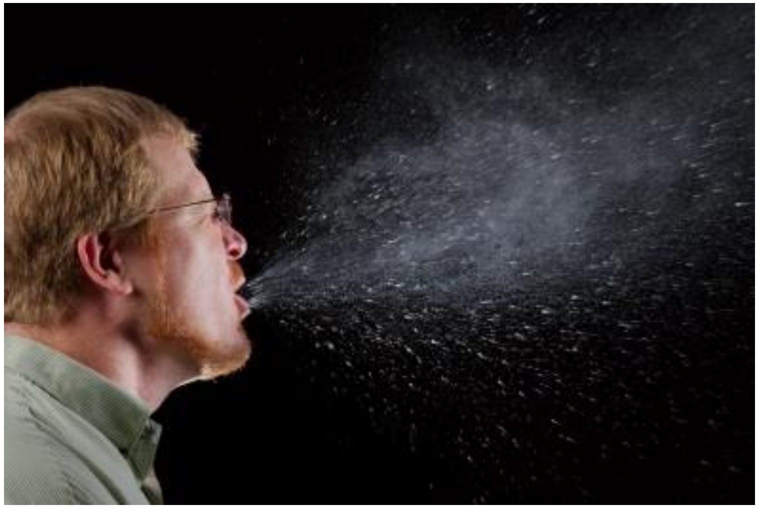

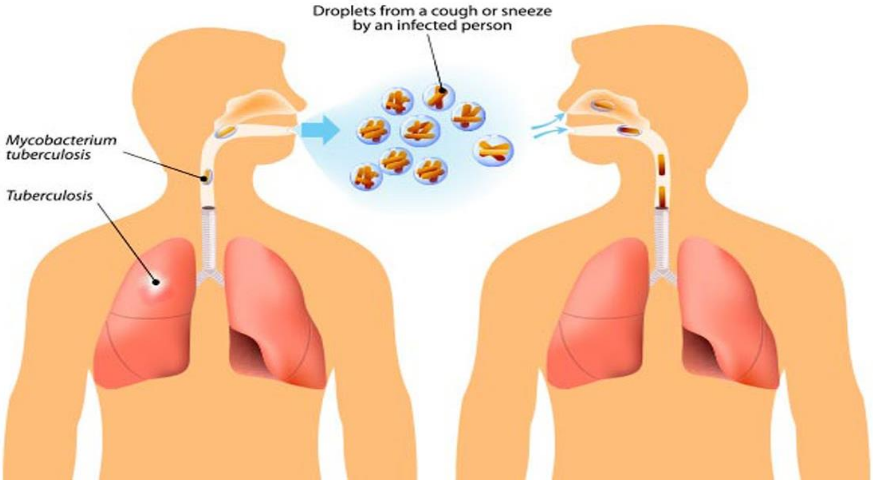

Who can get tuberculosis?

Everyone who have been in contact with a person with infectious lung tuberculosis can get tuberculosis

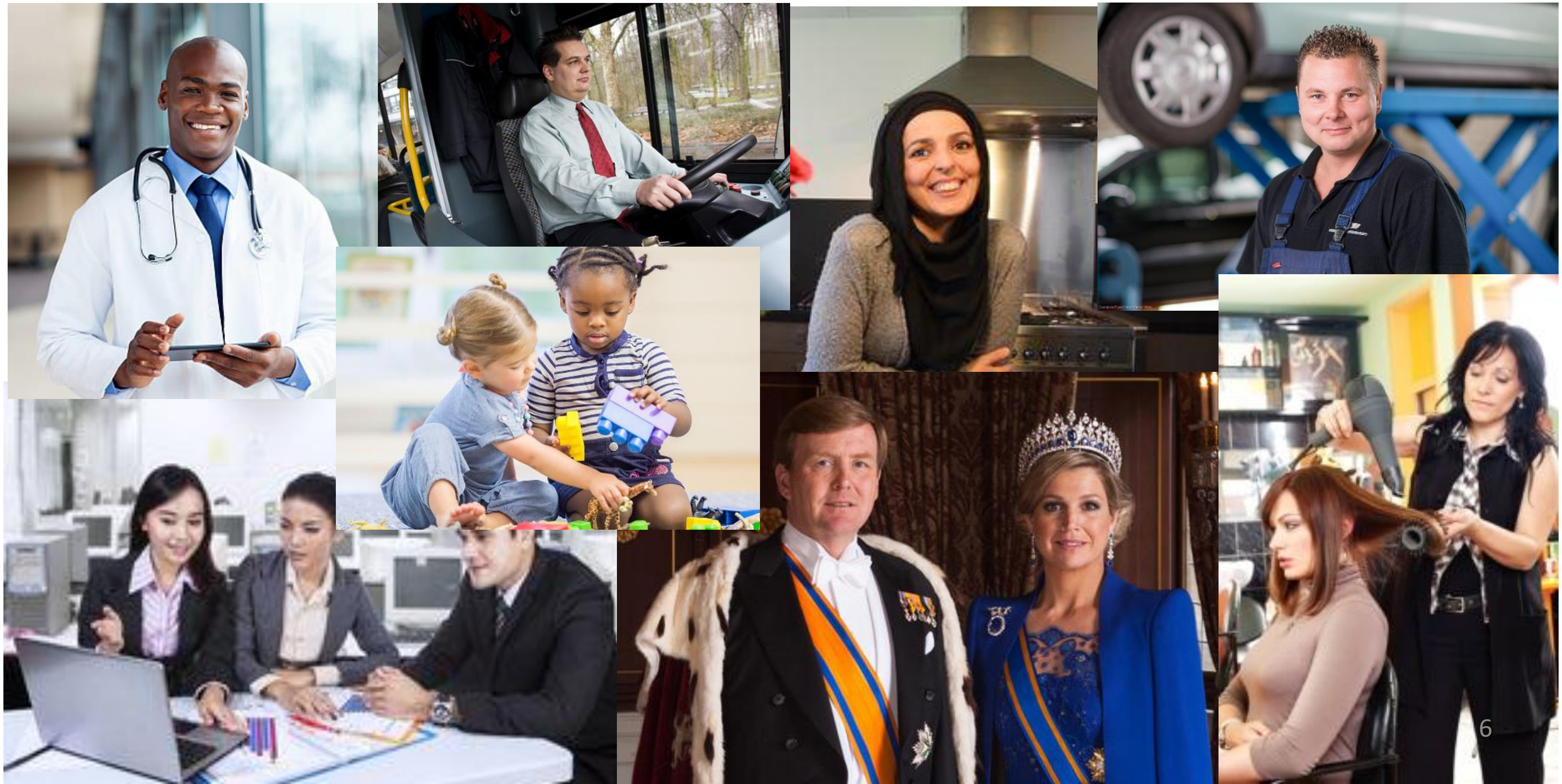

Quiz

How can tuberculosis be transmitted?

Tuberculosis in the world

- 10.4 million new TB cases each year
- 1.7 million deaths every year

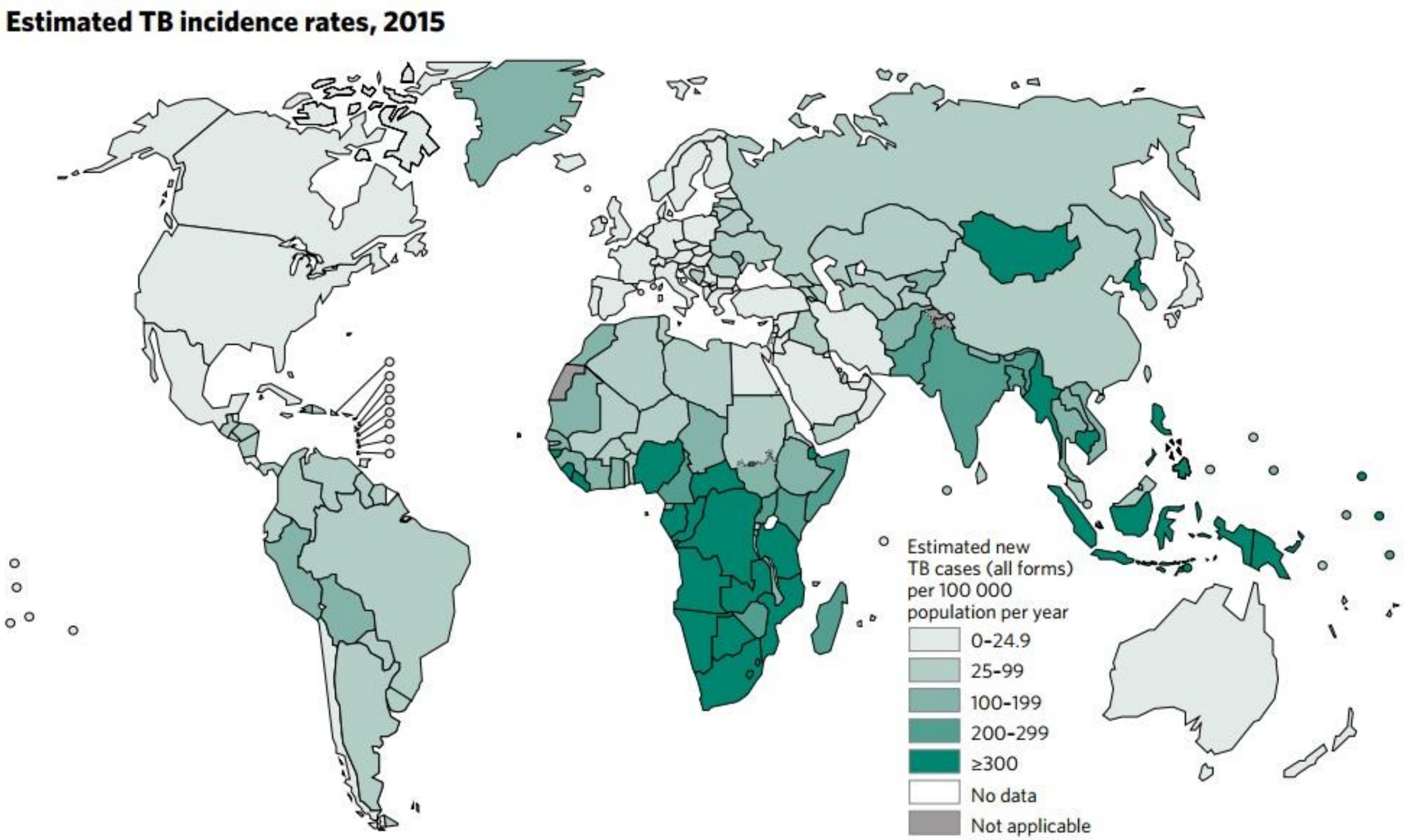

Tuberculosis in the Netherlands

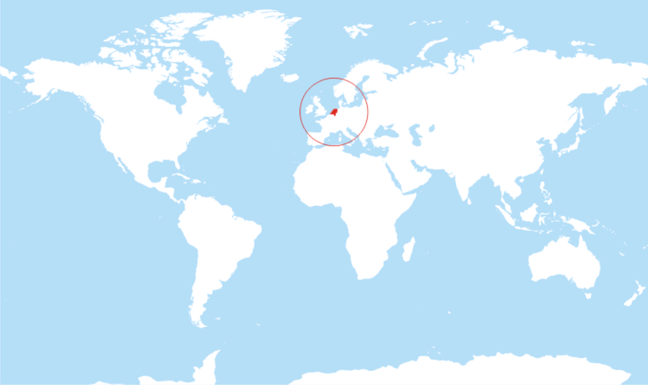

In 1951
→ 110 / 100.000 population

- Medication
- Improved hygiene & social welfare
- Good tuberculosis care

In 2016
→ 5.0 /100.000 population

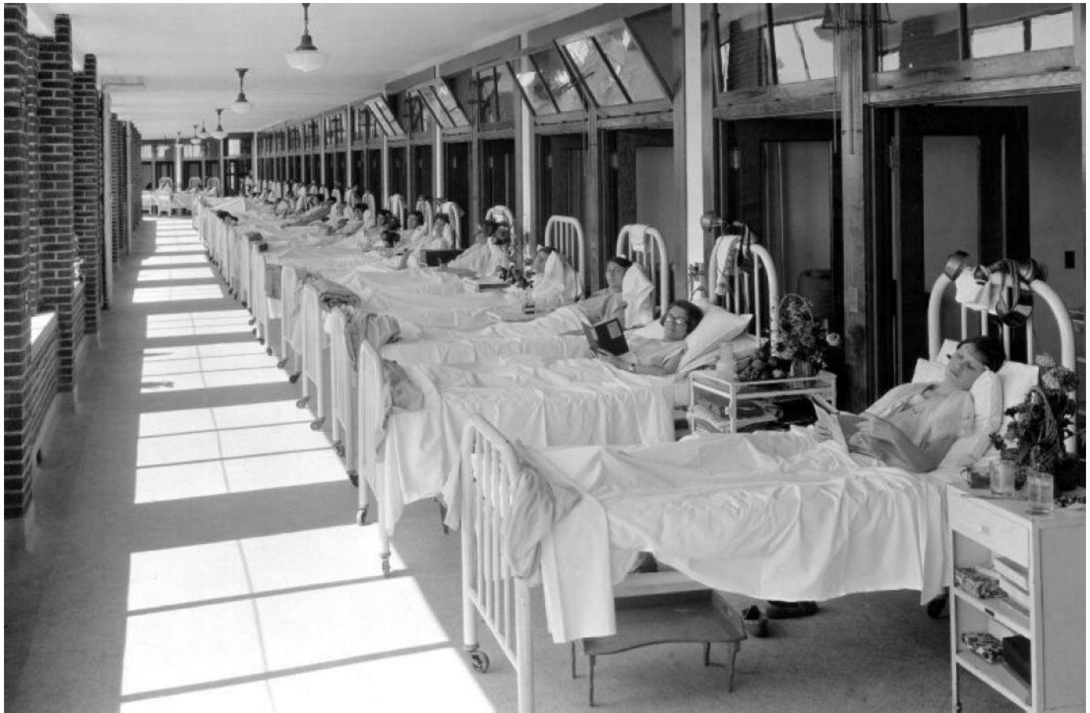

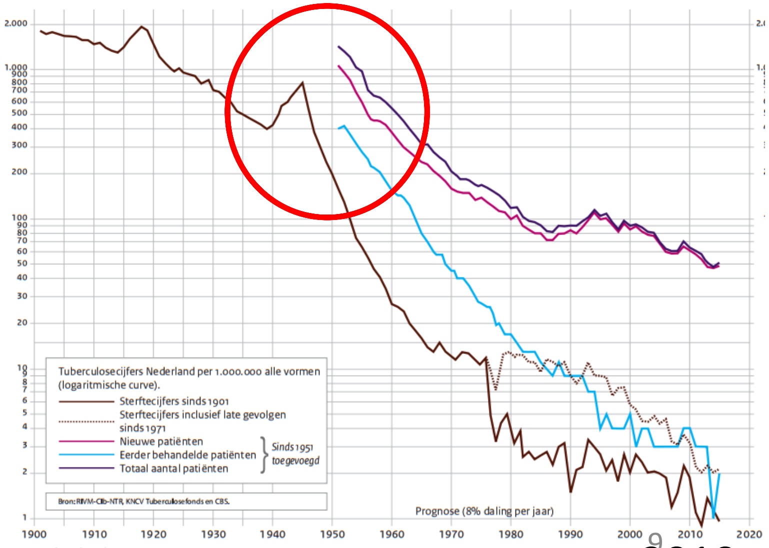

1900 2010

Tuberculosis screening in the Netherlands

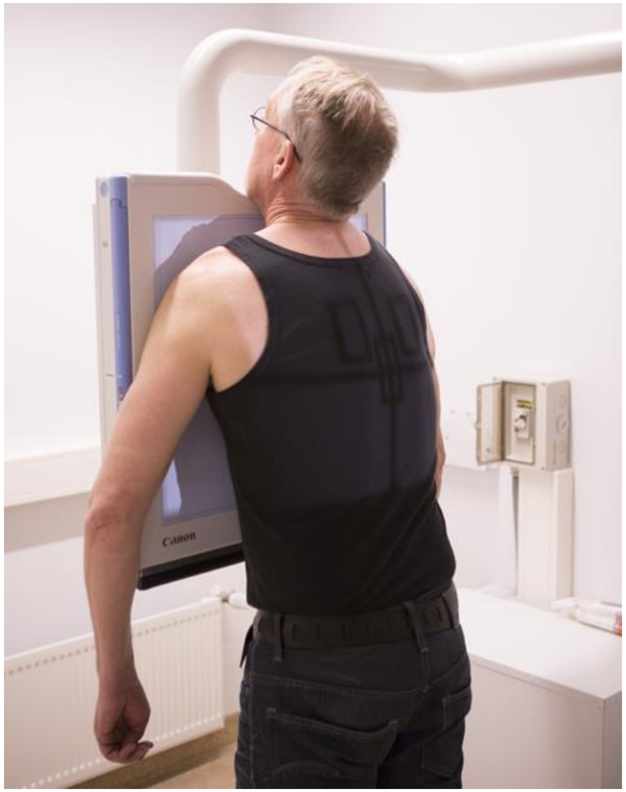

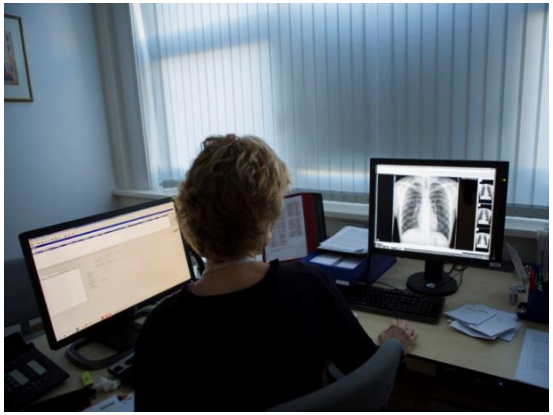

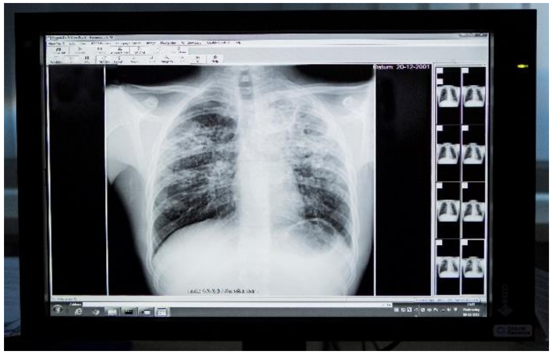

ENTRY SCREENING
All migrants from countries where tuberculosis is still prevalent

FOLLOW-UP SCREENING
All migrants from countries where tuberculosis is still **HIGH** prevalent

What happens if tuberculosis is found?

Everyone with tuberculosis will receive treatment with medication

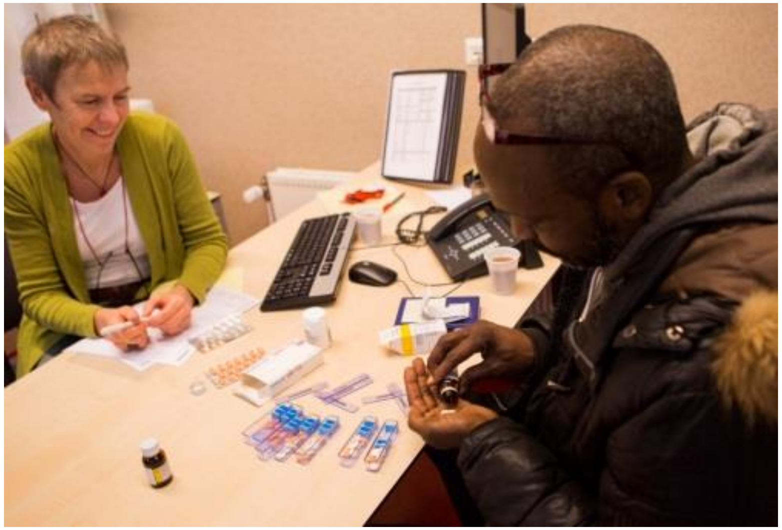

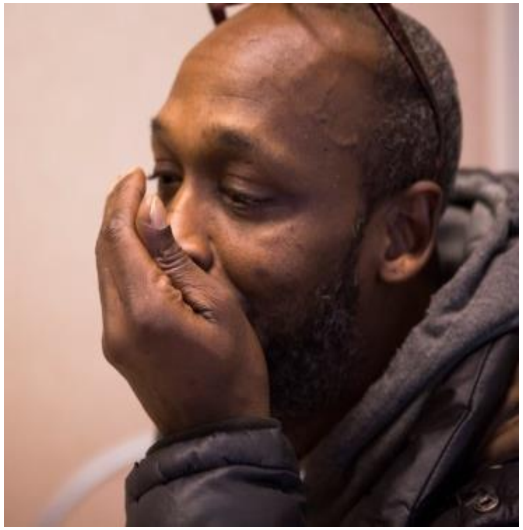

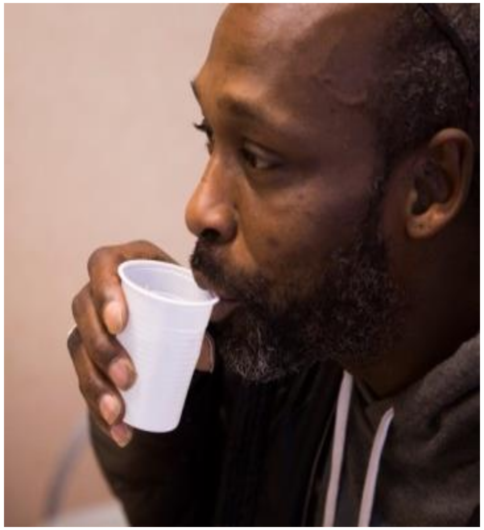

- Medication for 6 months
- Medication every day
- The tuberculosis nurse will give support

Film
TB: the Real Story

https://www.youtube.com/watch?v=89XaAFbaFwU (Tigrinya version)
https://www.youtube.com/watch?v=a2vAmk8aFjQ (English version)

Can tuberculosis be prevented?

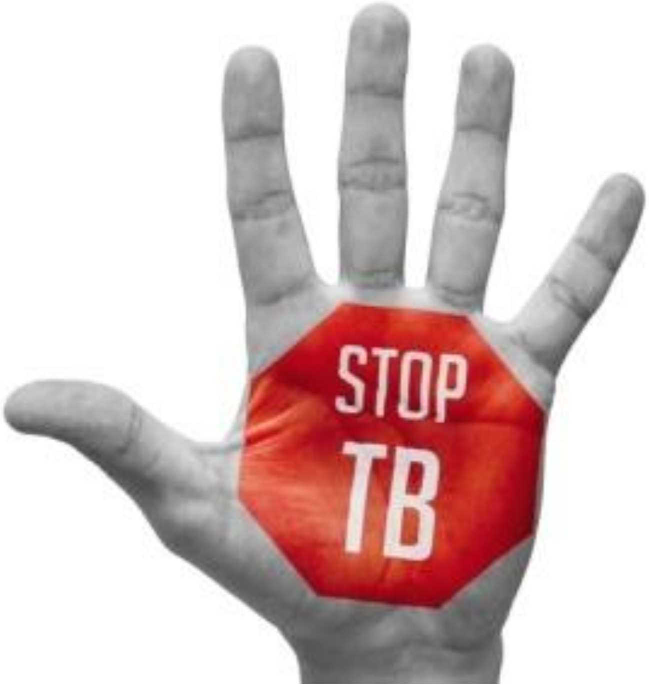

YES

How can tuberculosis be prevented?

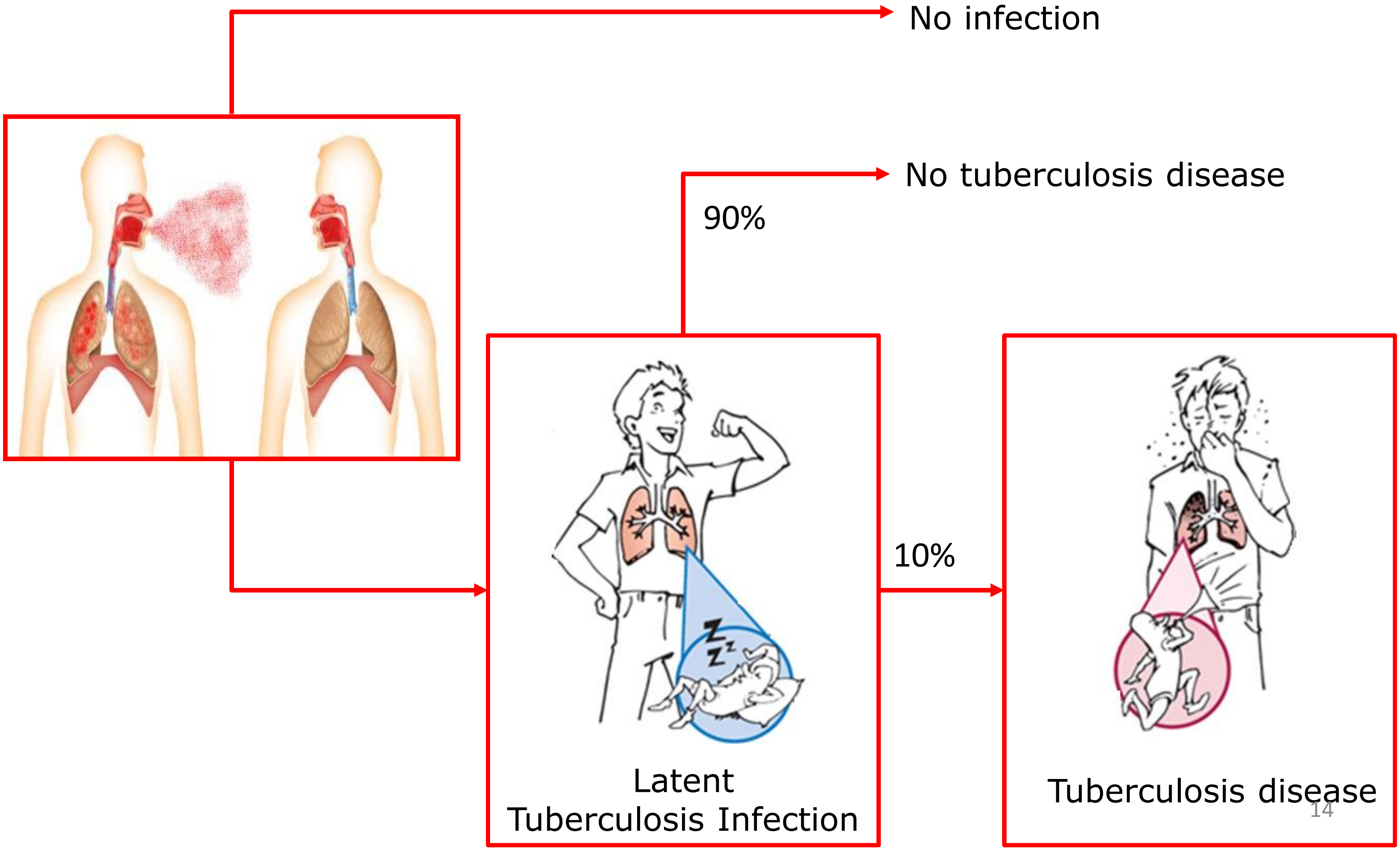

Latent Tuberculosis infection VS. Tuberculosis disease

Tuberculosis bacteria are sleeping in my body, but could wake up in the future	Tuberculosis bacteria are active, they make me sick
I am not sick, I do not have symptoms	I am sick, I have symptoms
My chest Xray is **normal**	My chest Xray is **abnormal**
I CANNOT spread tuberculosis bacteria	I CAN spread tuberculosis bacteria
Tuberculin Skin Test / Blood test is POSITIVE	Tuberculin Skin Test / Bloodtest is POSITIVE

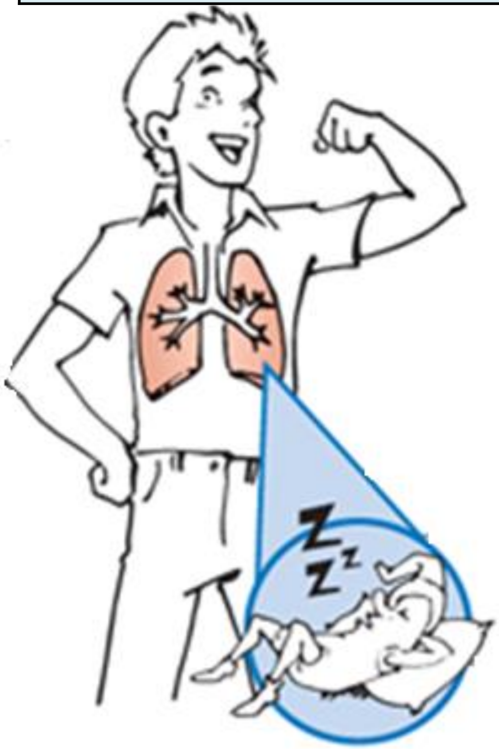

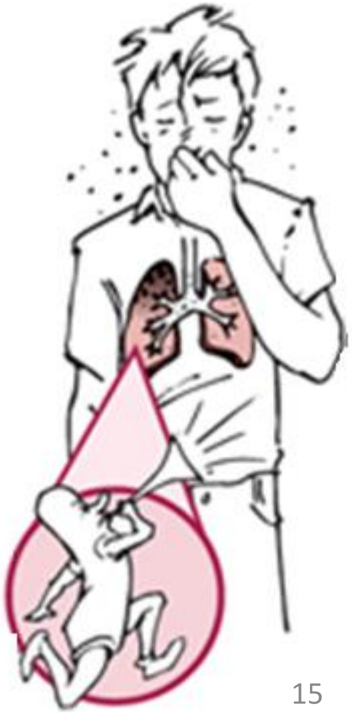

Who has highest risk of tuberculosis disease?

All persons with recent infection (infection < 2.5 years)

But also.....

Children

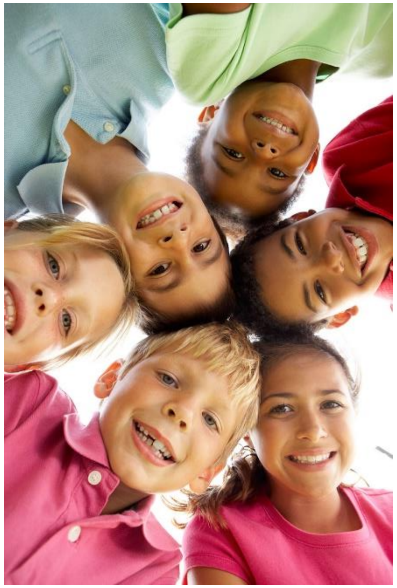

Drug and Alcohol abusers

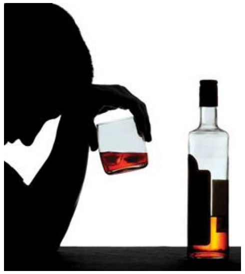

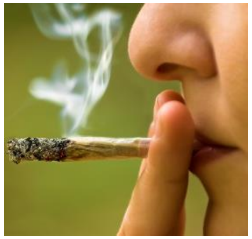

Persons with a weak immune system

- HIV/AIDS
- Diabetes
- Medication
- Cancer

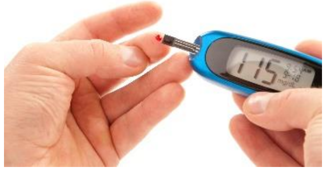

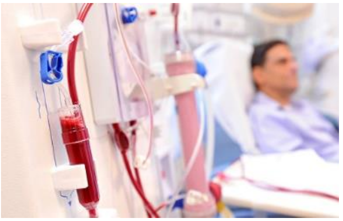

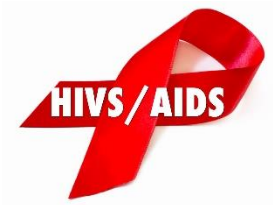

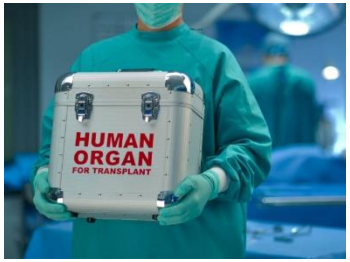

Quiz

Who has highest chance of getting tuberculosis disease?

How can we diagnose latent tuberculosis infection?

Tuberculin Skin Test

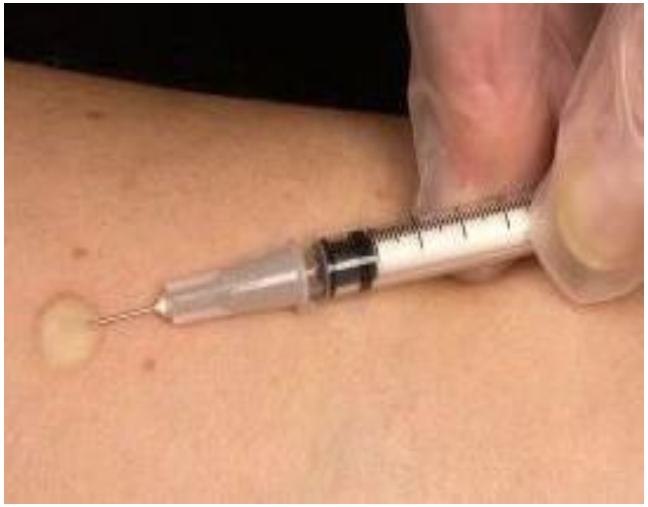

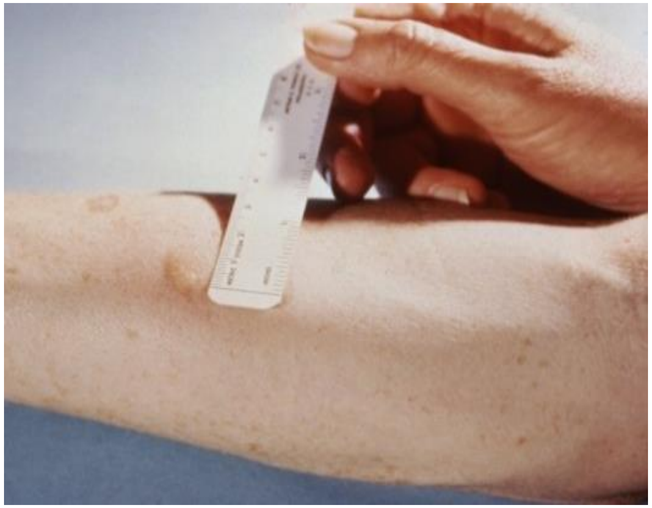

AND / OR

Blood test

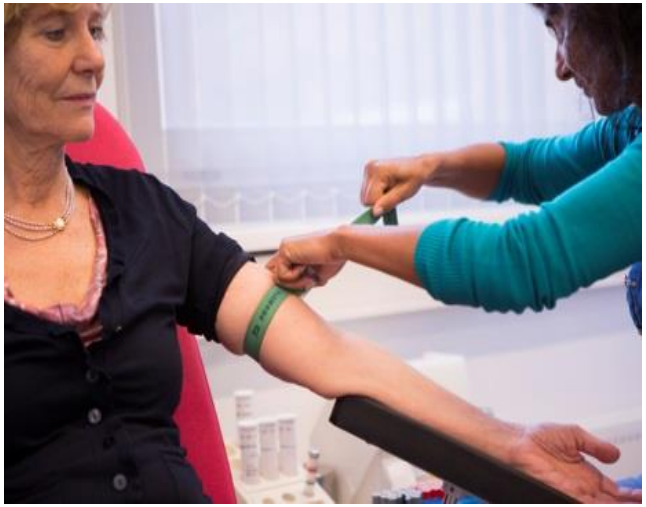

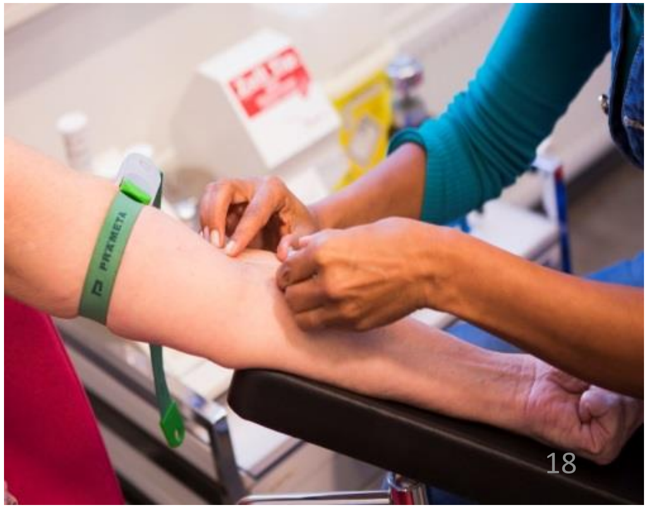

De tbc-bloedtest is positief: wat nu?

De dokter(sassistent) wil u graag verder helpen, doe daarom ook het vervolgonderzoek

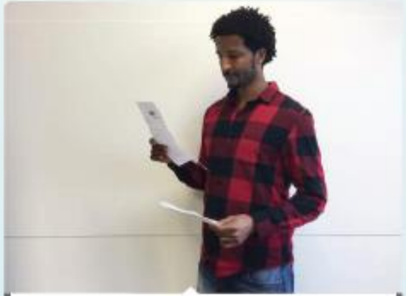

U krijgt een brief toegestuurd met de uitslag van de tbc bloedtest. Als de bloedtest positief is krijgt u een afspraak bij de dokter.

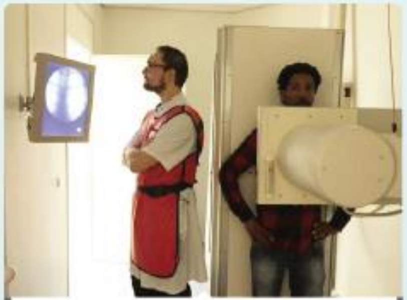

De dokter(sassistent) maakt een longfoto.

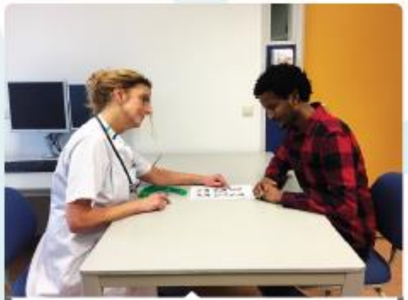

Op de longfoto ziet de dokter wat er aan de hand is en wat u moet doen.

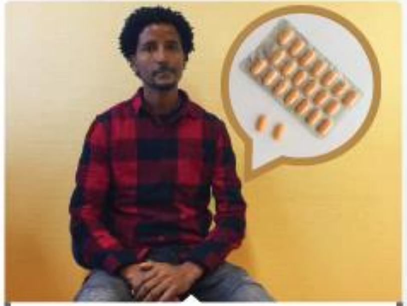

U heeft de tbc-bacterie

U merkt daar meestal niets van. U bent niet besmettelijk voor anderen.
De dokter adviseert u medicijnen te nemen. Zo voorkomt u dat u ziek wordt van tuberculose. En door medicijnen te nemen, maakt u anderen niet ziek.

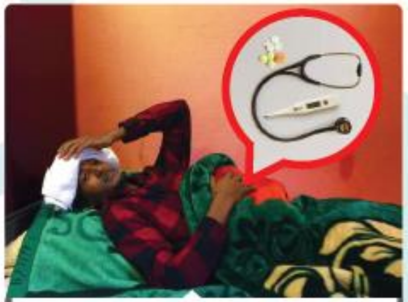

Longtbc

U heeft tbc. U kunt klachten hebben zoals veel hoesten, vermoeidheid en vermageren.
U bent vaak besmettelijk voor anderen.
De dokter behandelt u met medicijnen.

Treatment Latent Tuberculosis Infection

What?	tablets
How often?	every day
How long?	3 months

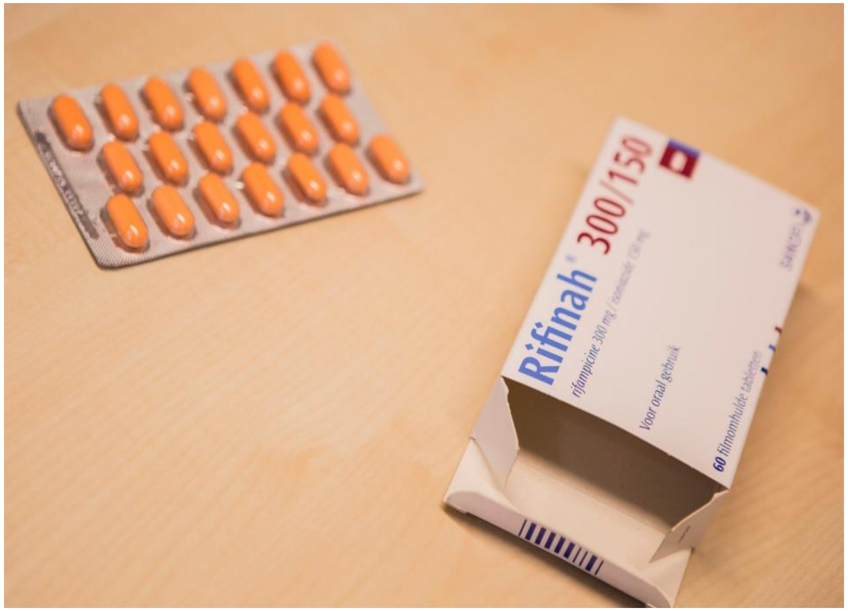

Quiz

Latent tuberculosis infection

Why should you do the test for latent tuberculosis infection?

- Horn of Africa there is still a lot of tuberculosis
- Eritreans in the Netherlands develop a lot of tuberculosis

- X- ray can only detect active tuberculosis in the lungs
- Tuberculosis bacteria can sleep for a long time (>10 years)
- This sleeping bacteria can only be identified through the blood test

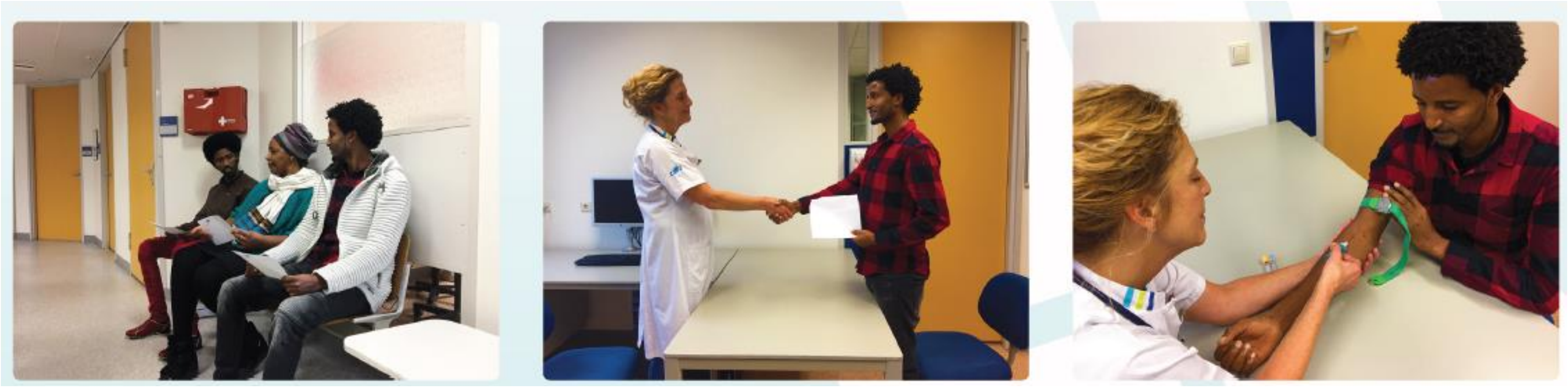

Upcoming tuberculosis screening

The TB ENDPoint research project offers a unique opportunity

- Organized by your GGD
- Free screening
- Free treatment

Bring family or friends!

All persons born in Eritrea/Ethiopia who have been in the Netherlands less than 10 years are welcome!

Bring your **identification card** and your **insurance card**

ጥዕናዊ ኣስተምህሮ

መልከፍቲ ባክተርያ ዓባይ ሰዓል፡- *መርመራን መከላኸሊ ፍወሳን*

KNCV TUBERCULOSISFOUNDATION

To eliminate TB

ሕማም ዓባይ ሰዓል እንታይ'ዩ?

- ለበዳዊ ሕማም
- ብባክተርያ ዝመጽእ

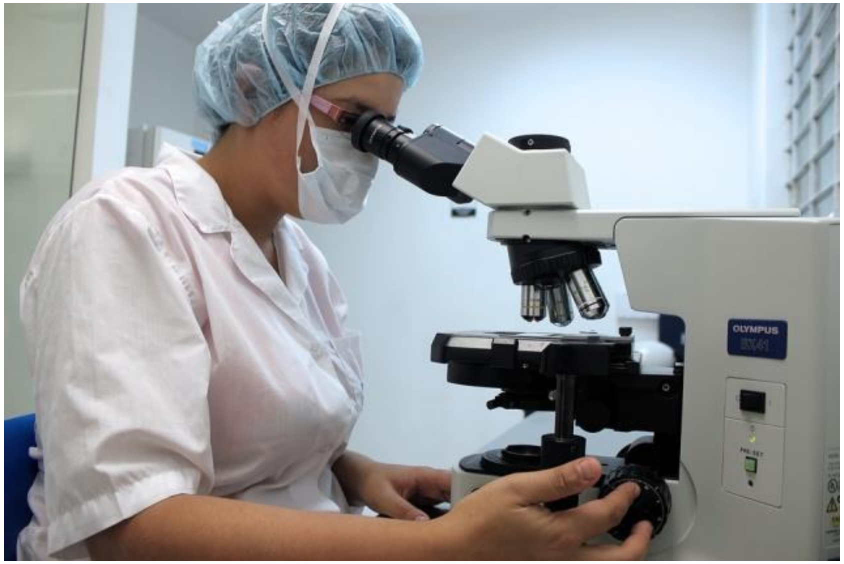

ማይክሮስኮፕ

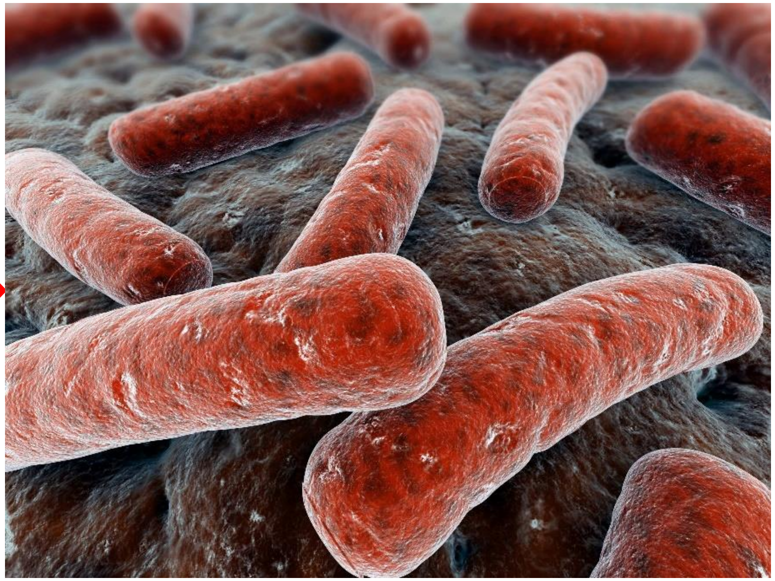

ማይኮ-ባክተሪዩም ቱበርኩሎሲስ

ዓባይ ሰዓል ኣየናይ ክፍሊ ኣካላት የጥቅዕ?

ዓባይ ሰዓል ንዝተፈላለየ ክፍሊ ኣካላት
ከጥቅዕ ይኽእል

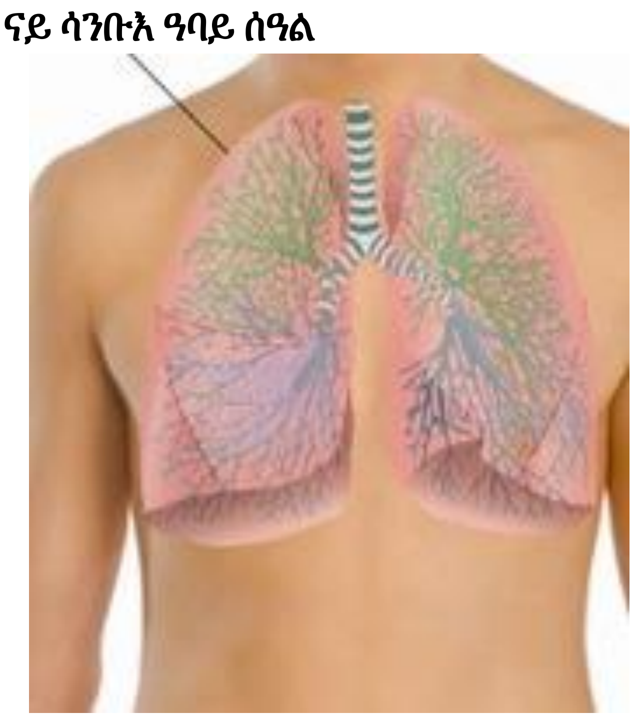

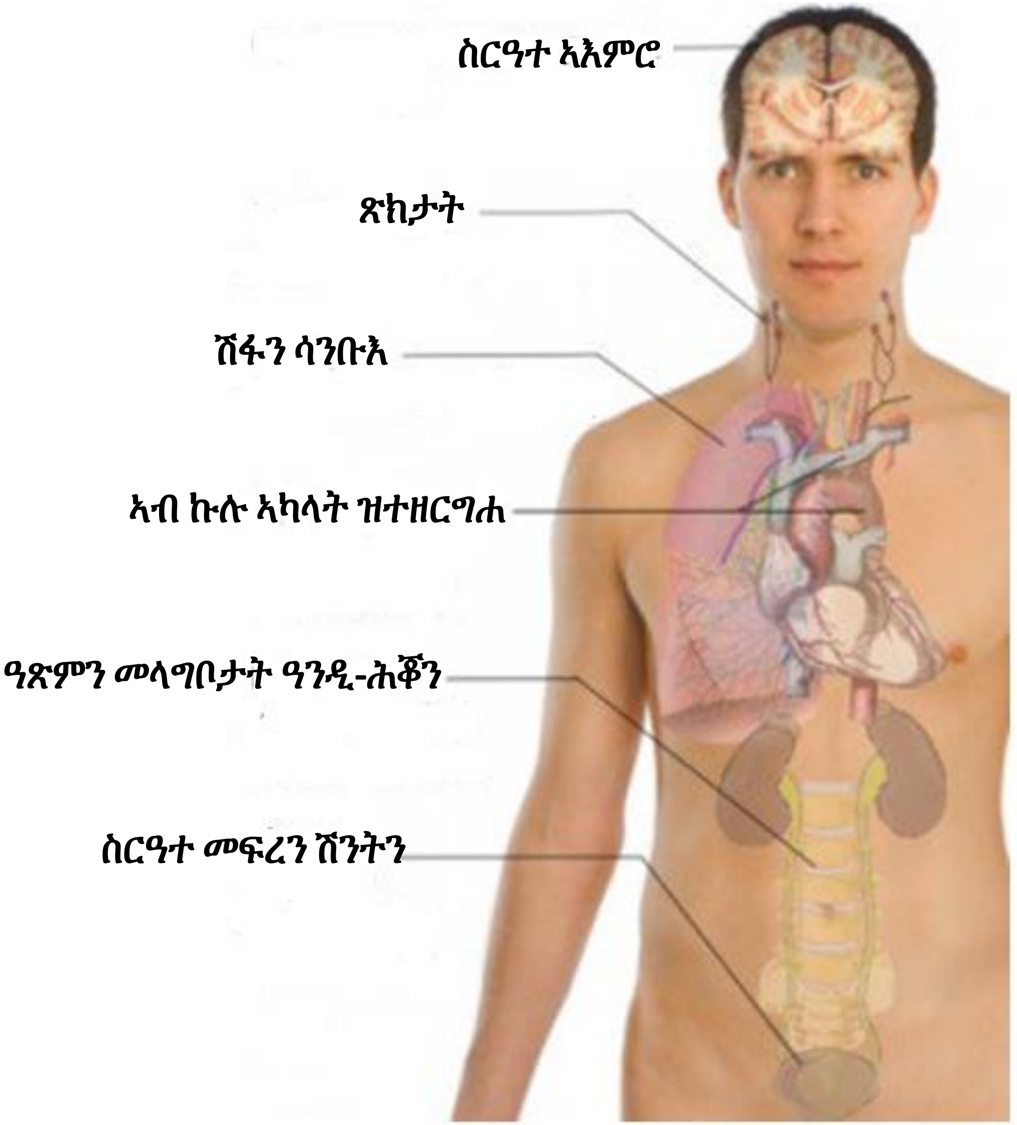

ምልክታት ሕማም ዓባይ ሰዓል እንታይ እዮም?

ሰዓል

ረስኒ

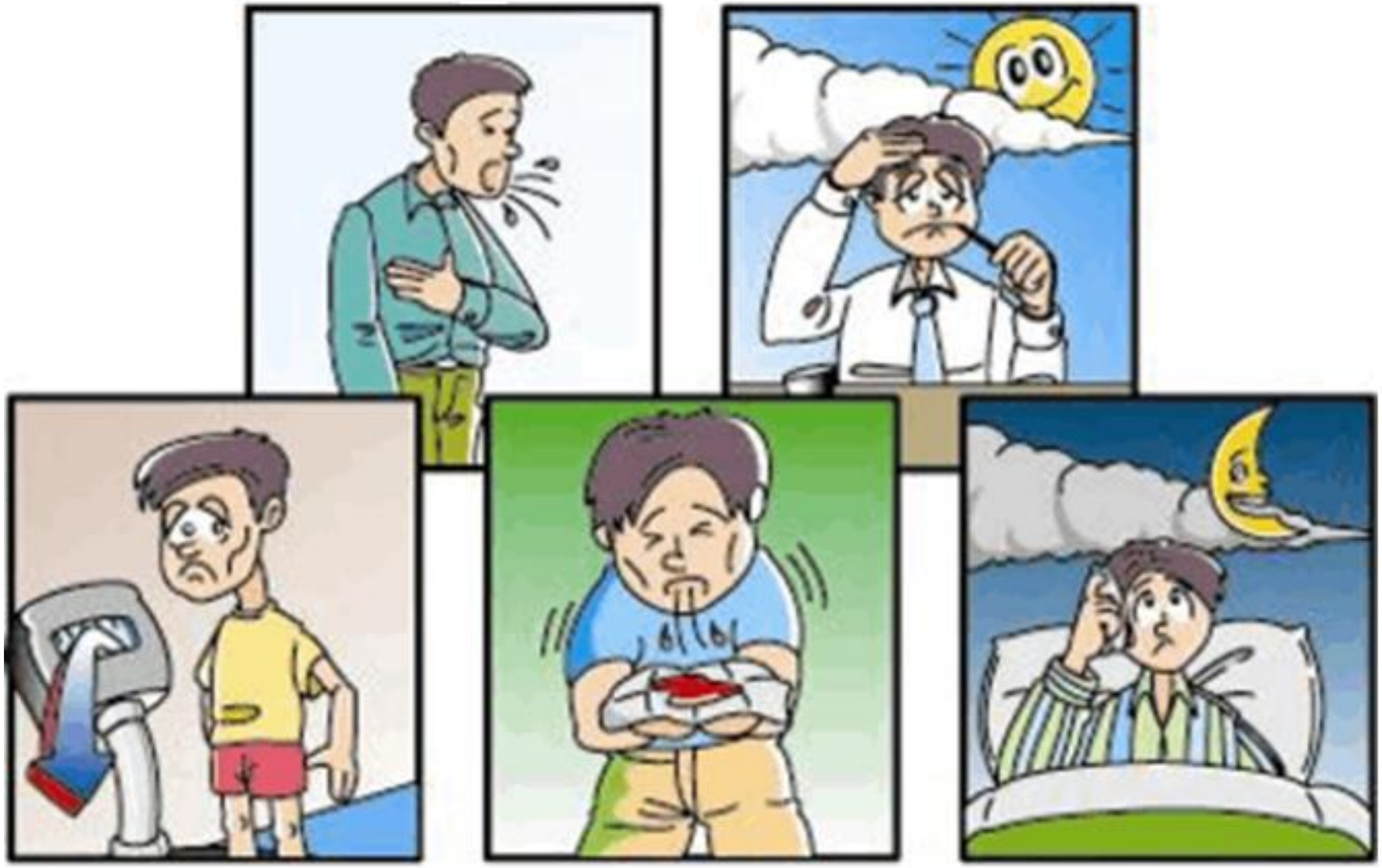

ምዛን ምጉዳል

ደም ዝተሓወሶ ዓኽታ

ረሃጽ ለይቲ

ዓባይ ሰዓል ብኸመይ ክለኽፈካ ይኽእል?

ዓባይ ሰዓል ብንፋስ ይመሓላለፍ

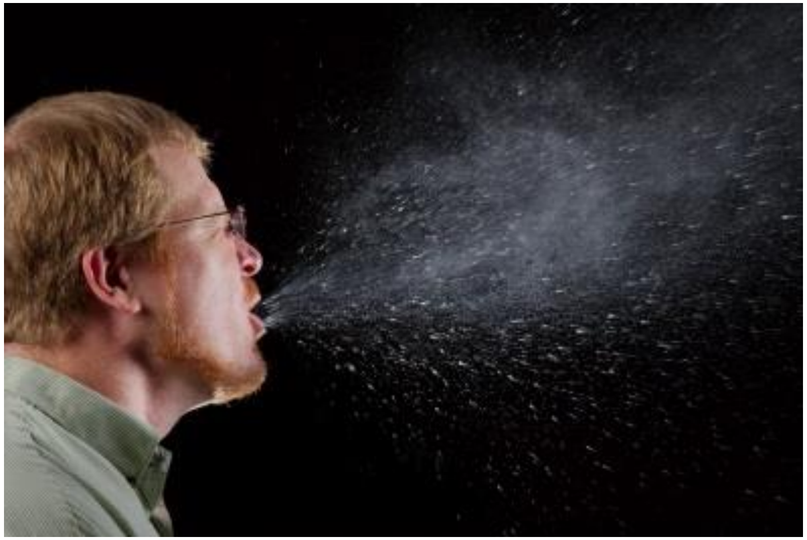

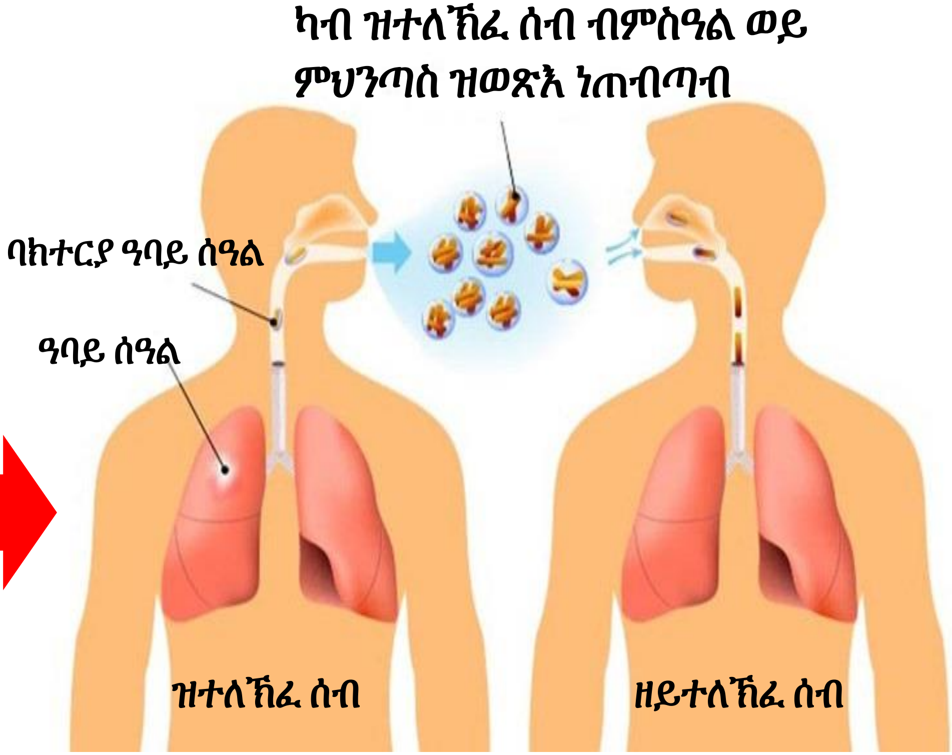

ብዓባይ ሰዓል መን ክልከፍ ይኽእል?

ምስ ተላባዒ ናይ ሳንቡእ ዓባይ ሰዓል ዘለዎ ሰብ ዝተራኸበ ዝኾነ-ሰብ ክልከፍ ይኽእል!

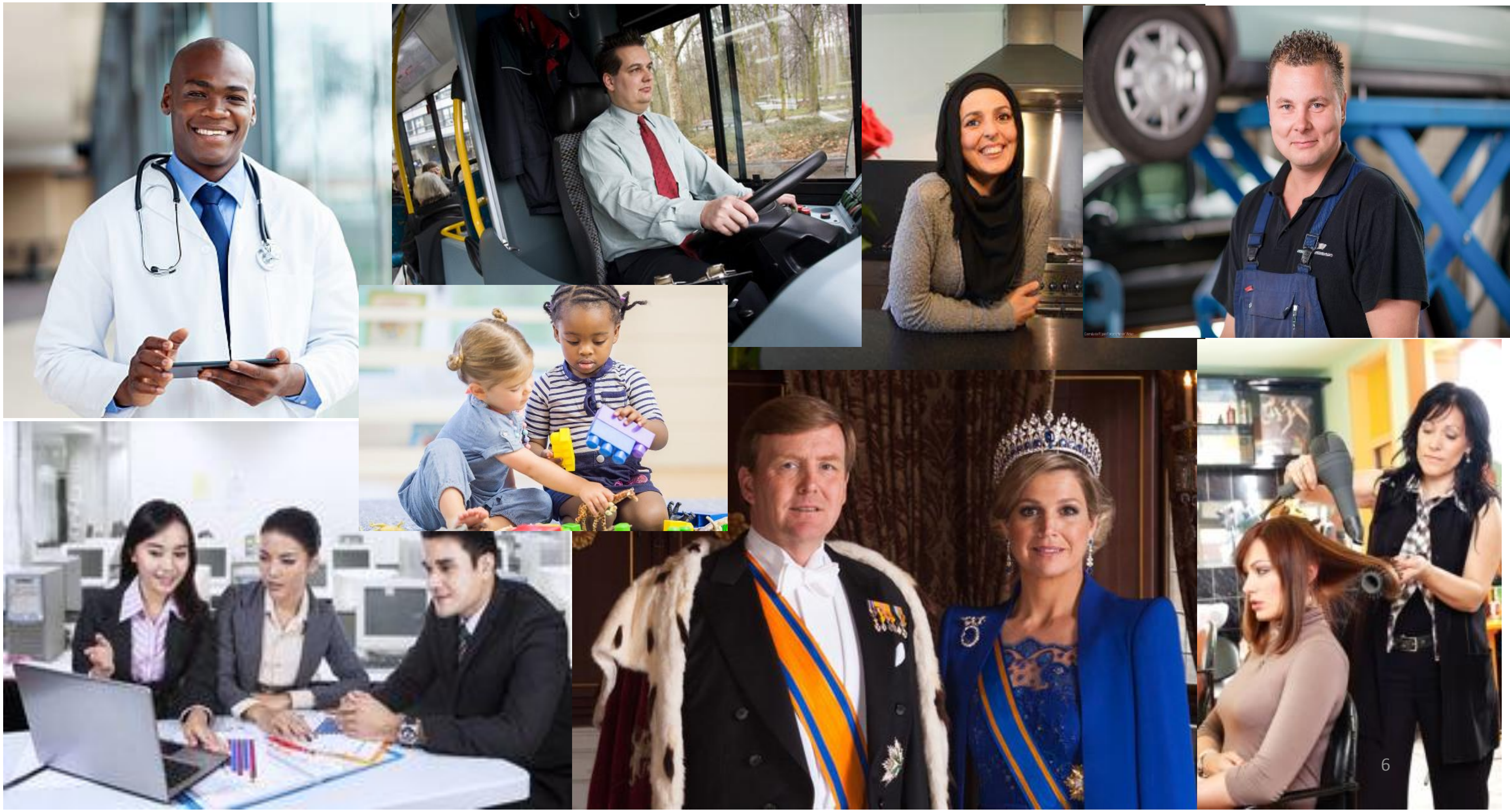

ሕቶ

ሕማም ዓባይ ሰዓል ብኸመይ ክመሓላለፍ ይኽእል?

ዓባይ ሰዓል ብዓለም ደረጃ

- 10.4 ሚልዮን ዓመታዊ ሓድሽ ክስተታት ዓባይ ሰዓል
- 1.7 ሚልዮን ዓመታዊ ናይ ሞት ብዝሒ

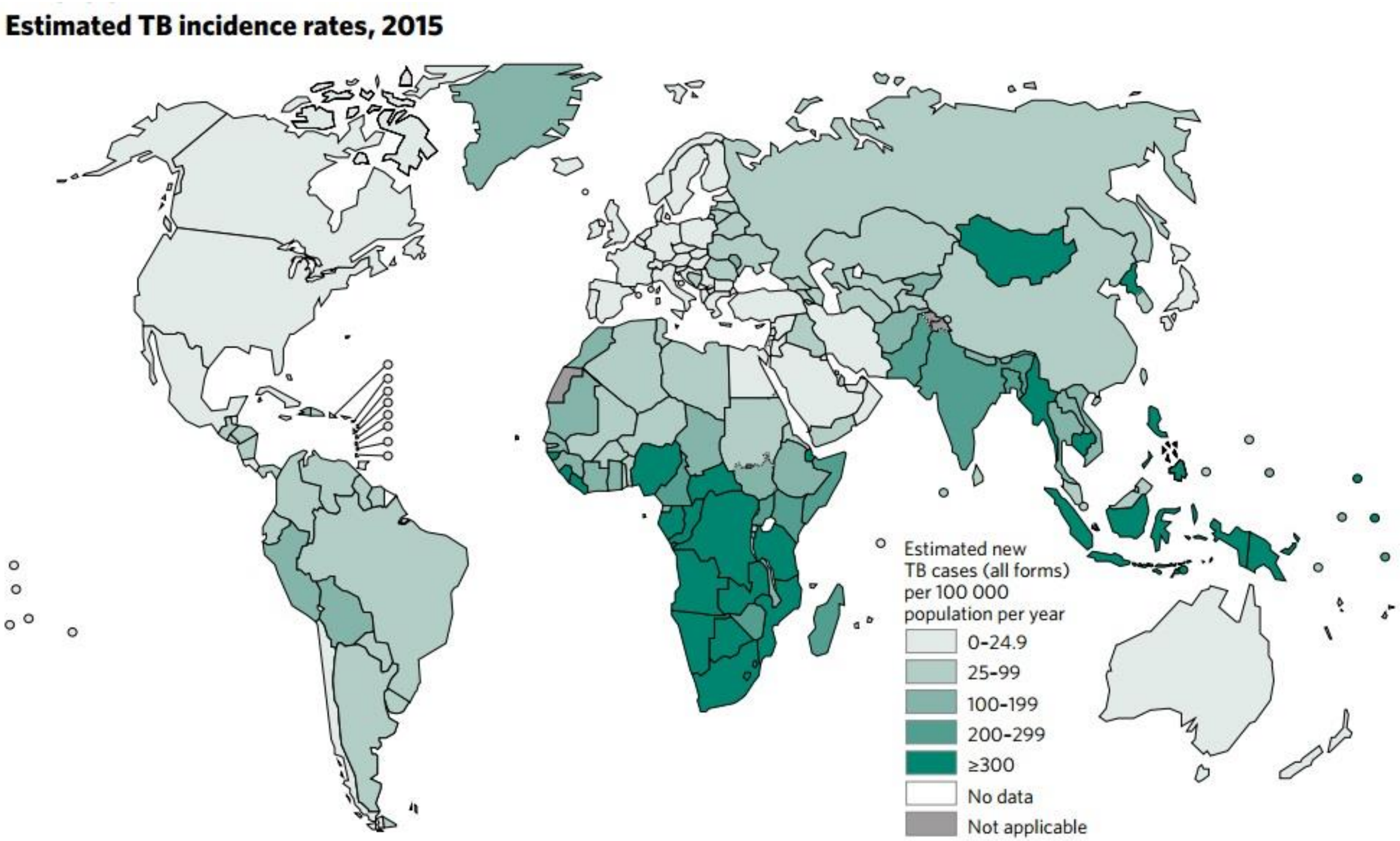

ዓባይ ሰዓል ኣብ ሃገረ ኔዘርላንድ

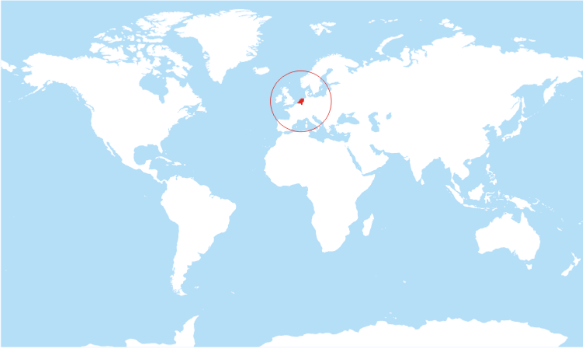

In 1951
→ 110 / 100.000 ብዝሒ ህዝቢ

- ❖ፍወሳ
- ❖ዝተማሓየሸ ስነ-ጥዕናዊ ጽሬትን ማሕበራዊ ድሕነትን
- ❖ዝተማሓየሸ ናይ ዓባይ ሰዓል ኣተኣላልያ

In 2016
→ 5.0 /100.000 ብዝሒ ህዝቢ

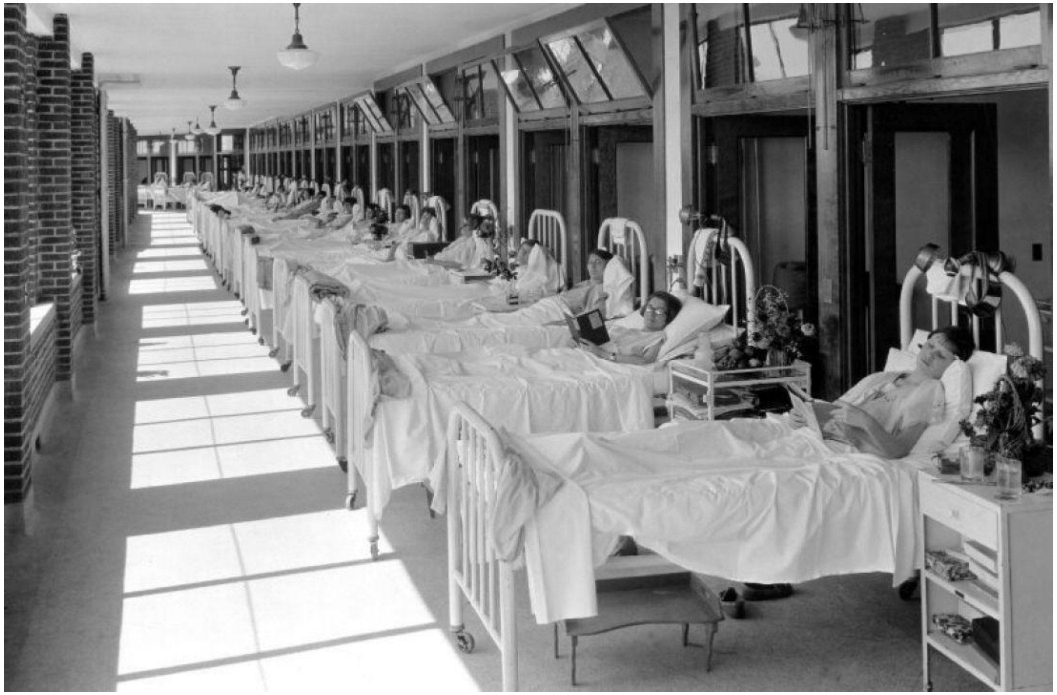

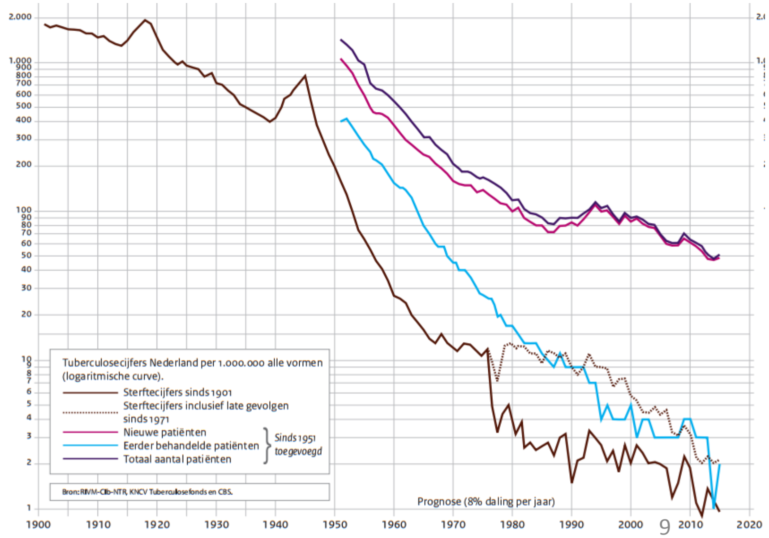

መርመራ ዓባይ ሰዓል ኣብ ሃገረ ኔዘርላንድ

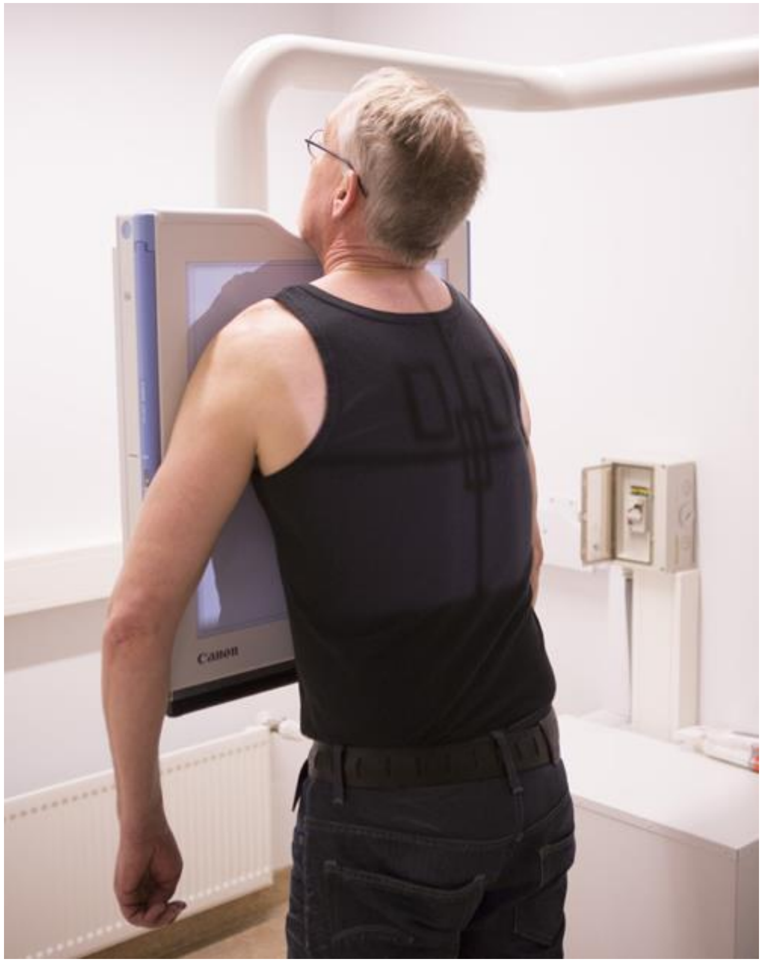

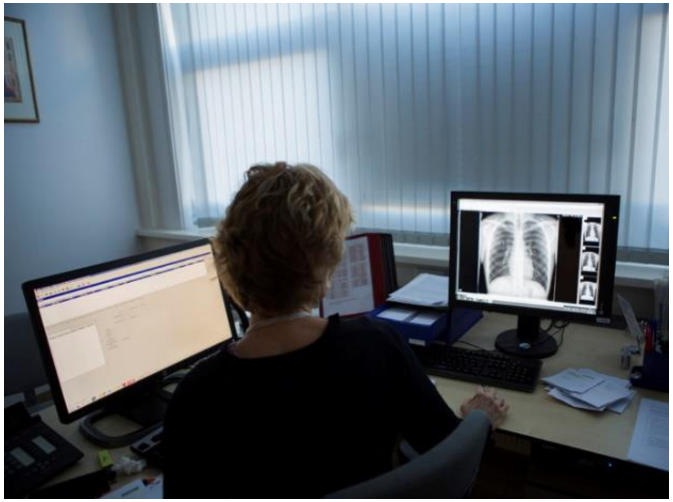

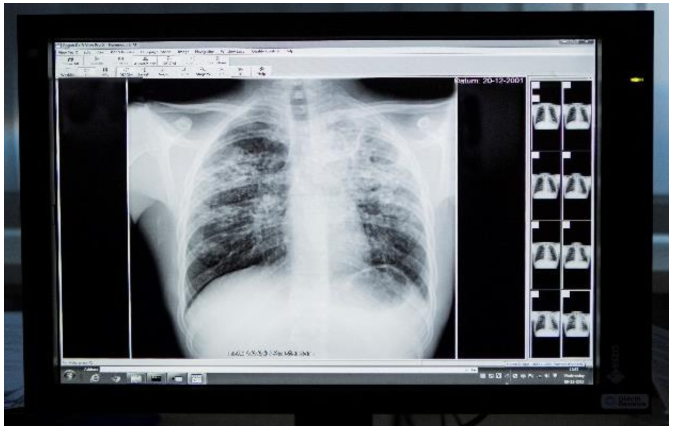

መርመራ ኣብ ምእታው
ኩሉም ስደተኛታት ካብተን ተርእዮ ዓባይ ሰዓል ዝኸሰተለን ሃገራት ዝመጹ

ናይ ምክትታል መርመራ
ኩሉም ስደተኛታት ካብተን ተርእዮ ዓባይ ሰዓል **ብዝለዓለ** ዝኸሰተለን ሃገራት ዝመጹ

ሕማም ዓባይ ሰዓል እንተተረኺቡ ዝስዕብ መስርሕ እንታይ'ዩ?

ኩሉ ዓባይ ሰዓል ዝተረኸቦ ሰብ ፡ ብመድሃኒት ዝውሰድ ፍወሳ ይወሃቦ

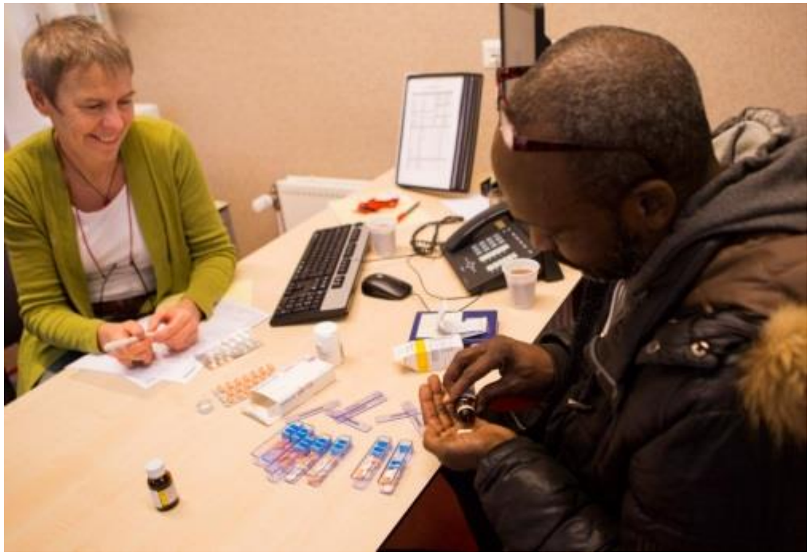
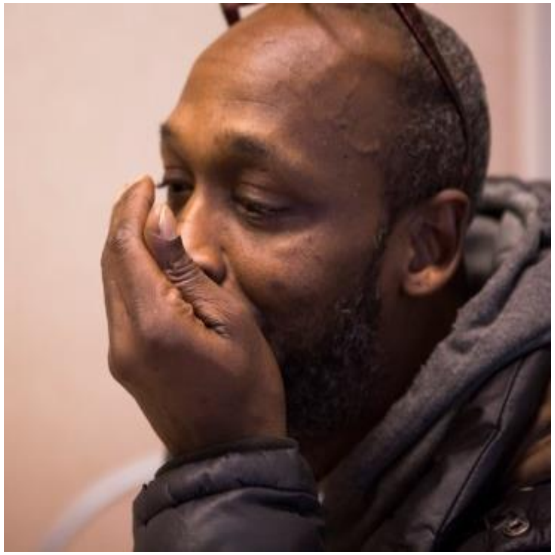
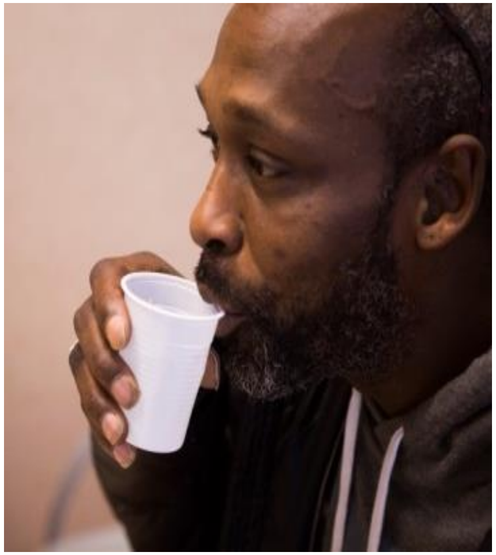

- ናይ 6 ወርሒ መድሃኒት
- መዓልታዊ ዝውሰድ መድሃኒት
- ናይ ዓባይ ሰዓል ነርስ ሓገዝ ትልግስ

ፊልም

ሕማም ዓባይ ሰዓል፡ ሓቀኛ ዛንታ

https://www.youtube.com/watch?v=89XaAFbaFwU (Tigrinya version)

https://www.youtube.com/watch?v=a2vAmk8aFjQ (English version)

ዓባይ ሰዓል ክንክላኸሎ ወይ ክግታእ ይከኣል'ዶ?

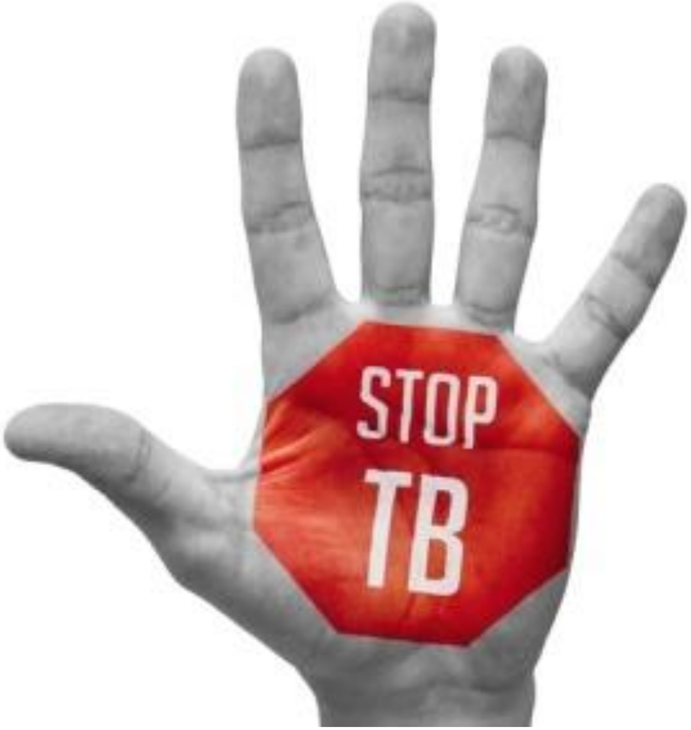

እወ

ዓባይ ሰዓል ብኸመይ ክግታእ ይኽእል?

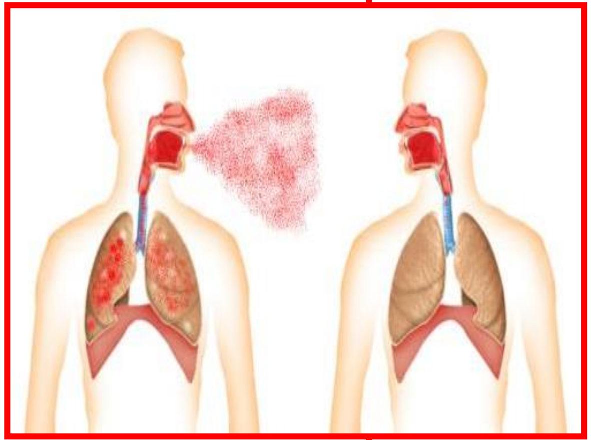

መልከፍቲ ኣየጋጥመን

90%

ሕማም ዓባይ ሰዓል የለን

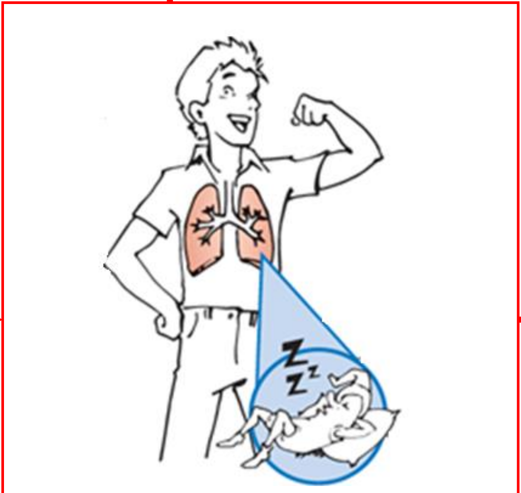

መልከፍቲ ባክተርያ ዓባይ ሰዓል

10%

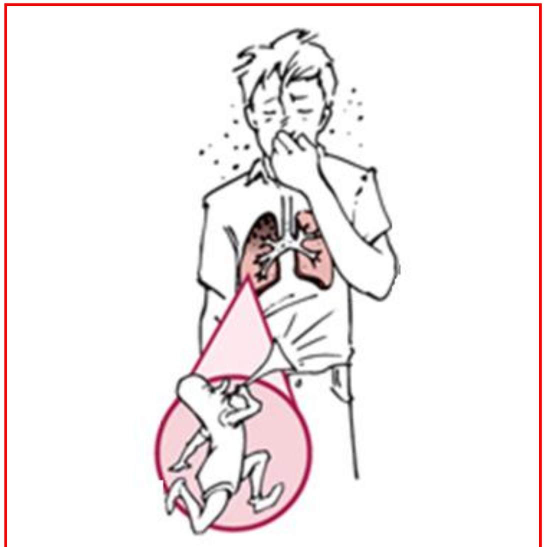

ሕማም ዓባይ ሰዓል

መልከፍቲ ባክተርያ ዓባይ ሰዓል ምስ ሕማም ዓባይ ሰዓል ክነጻጸር

ዘይንጡፍ ባክተርያ ዓባይ ሰዓል ኣብ ኣካላተይ ደቂሱ ይርከብ እንተኾነ ኣብ መጻኢ ንጡፍ ክኸውን ይኽእል'ዩ	ባክተርያ ዓባይ ሰዓል ንጡፋት ኮይኖም፡ ሕማም ኣኸቲሎምለይ ይርከቡ
ኣይሓመምኩን ዝኾነ ናይ ሕማም ምልክታት'ውን የብለይን	ሓሚመ ኣለኹ፡ ምልክታት ናይ ሕማም'ውን ኣለዉኒ
ናይ ሳንቡእ ስእሊ (X-ray) **ንቡር** ምልክት የርኢ	ናይ ሳንቡእ ስእሊ (X-ray) **ዘይንቡር** ምልክት የርኢ
ባክተርያ ዓባይ ሰዓል ከማሓላልፍ **ኣይክእልን**	ባክተርያ ዓባይ ሰዓል ከማሓላልፍ **ይክእል**
ናይ ዓባይ ሰዓል ናይ ቆርበት ወይ ድም መርመራ **ኣወንታ** ውጽኢት **የርኢ**	ናይ ዓባይ ሰዓል ናይ ቆርበት ወይ ድም መርመራ **ኣወንታ** ውጽኢት **የርኢ**

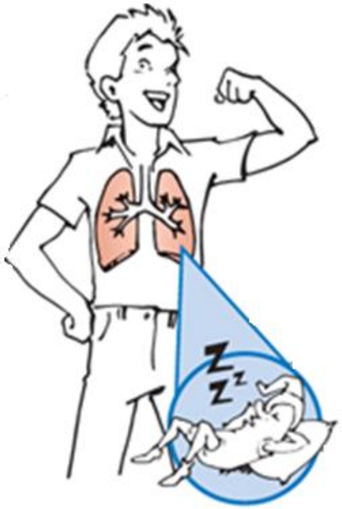

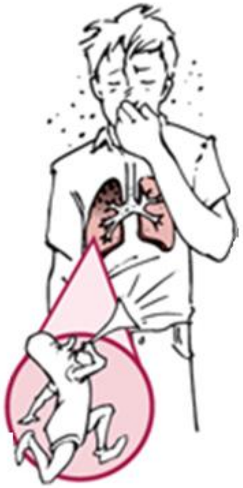

ዓባይ ሰዓል ናይ ምሕማም ዝለዓለ ተኽእሎ ዘለዎ መን'ዩ?

ኣብ ቀረባ እዋን ዝተለኽፈ ሰብ (እቲ መልከፍቲ ኣብ ውሽጢ ዝሓለፈ 2 ዓመትን ፈረቓን ምስዝኸውን)

ከምኡ'ውን........

ቆልዑ

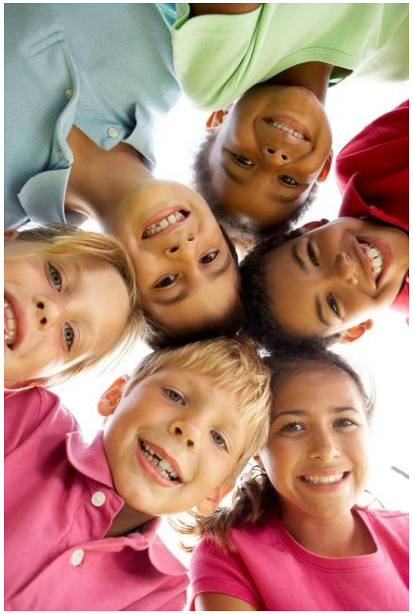

ናይ ኣልኮላዊ መስተን ዕጸ-ፋርስን ግዙኣት

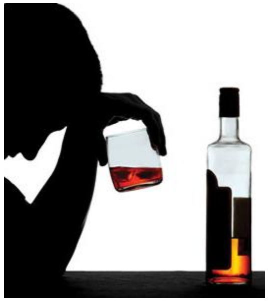

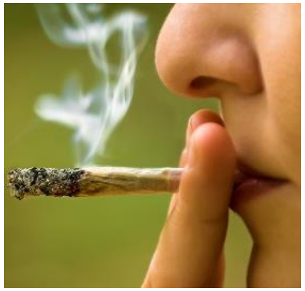

ትሑት ሕማም ናይ ምጽዋር ዓቕሚ ዘለዎም ሰባት

- ኤች ኣይ ቪ/ኤይድስ
- ሕማም ሽኮር
- መድሃኒት ዝጥቀሙ
- መንሽሮ

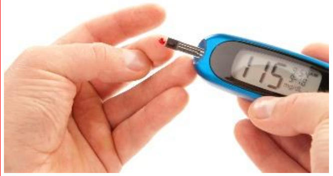

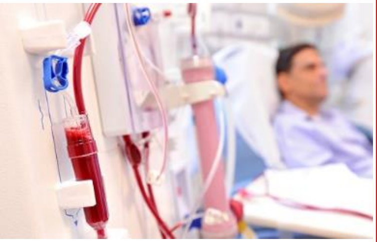

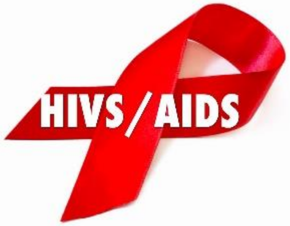

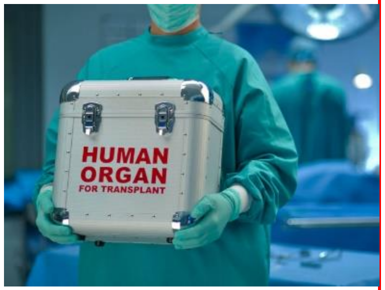

ሕቶ

ሕማም ዓባይ ሰዓል ናይ ምሕማም ዝለዓለ ተኽእሎ ዘለዎ መን’ዩ?

መልከፍቲ ባክተርያ ዓባይ ሰዓል ብኸመይ ክንምርምሮ ወይ ክነለልዮ ንኽእል?

ናይ ቆርበት መርመራ

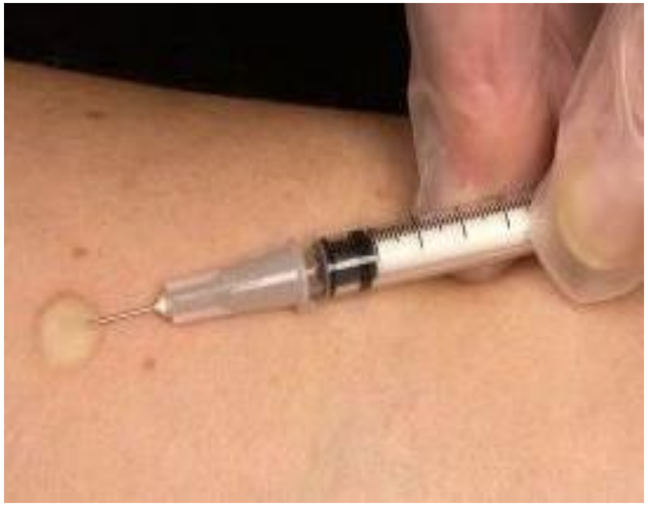

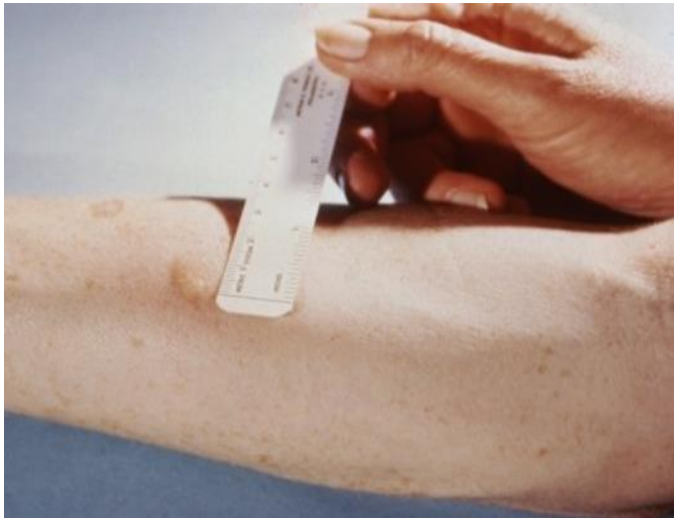

ወይ/ተወሳኺ

ደም መርመራ

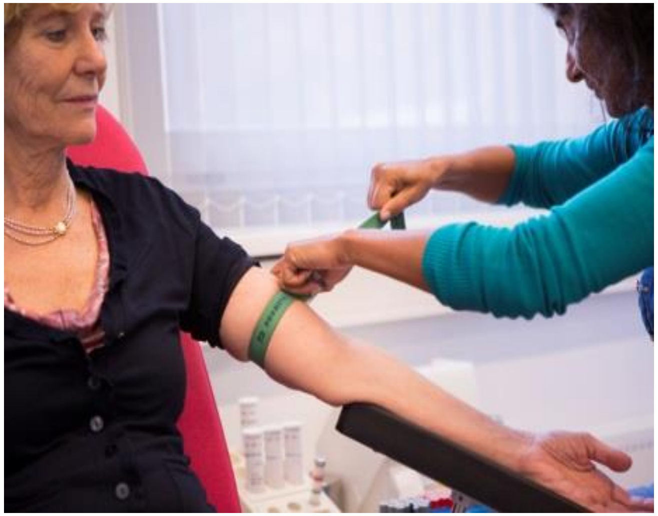

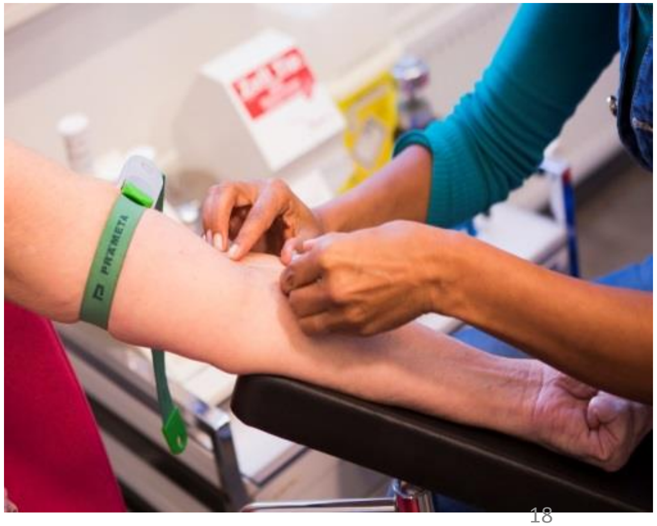

ውጽኢት ናይቲ ደም መርመራ ኣወንታ ኮይኑ ኣሎ፡ ዝስዕብ መስርሕ እንታይ'ዩ?

ሓኪም ክሕግዘካ ተዳልዩ ኣሎ፡
ስለዚ እቲ ዝስዕብ ተወሳኺ መርመራታት ግበር።

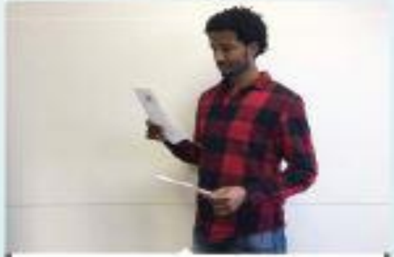

ውጽኢት ናይቲ ዝገበርካዮ መርመራ ዝሓዘ ደብዳቤ ክበጽሓካ እዩ። እቲ ውጽኢት ኣወንታ እንተኾይኑ፡ ናይ ሓኪም ቆጸራ ክግበረልካ እዩ።

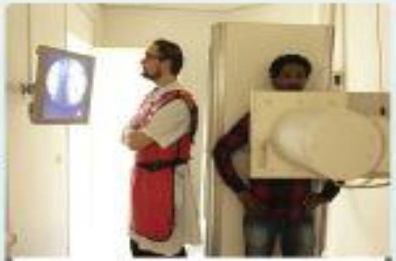

ሓኪም ናይ ሳንቡእ ስእሊ (x-ray) ይገብረልካ።

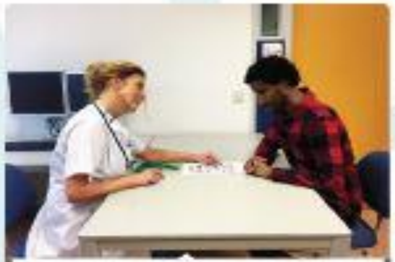

ሓኪም ነቲ ናይ ሳንቡእ ስእሊ (x-ray) ብምዕዛብ እንታይ ክትገብር ከምዘለካ ይሕብረካ።

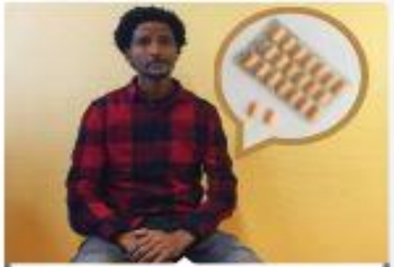

ባክተርያ ሕማም ዓባይ ሰዓል ኣብ ኣካላትካ ኣሎ

እቲ ባክተርያ ከምዝሓካ ወርሒ ምልክት ኣይረኣየካ። ንኻልኦት ሰባት ክተመሓላልፎ'ውን ኣይትኽእልን ኢኻ። እንተኾነ፡ ሓኪም ከነታትካ ናብ ሕማም ዓባይ ሰዓል ንኸይምዕበል ዝኽእል መድሃኒት ክትወስድ ትመኽረካ። ብዚ መገዲ ሕማም ዓባይ ሰዓል ካብ ምሕማም ክትገላገል ትኽእል። መድሃኒትካ ብግቡእ ብምውሳድ ንኻልኦት ካብ ምልካፍ'ውን ተድሕን።

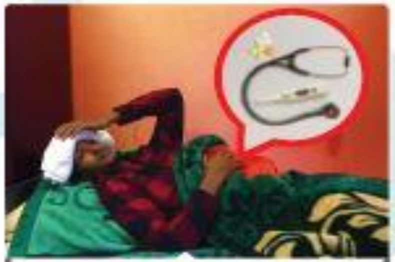

ናይ ሳንቡእ ሕማም ዓባይ ሰዓል

ሕማም ዓባይ ሰዓል ኣለካ፡ ሓሚምካ'ውን ኣለኻ። ከም ሰዓል፡ ድኻም ኣካላትን ምንካይ ሚዛን ሰብነትን ዝኣመሰሉ ምልክታት ሕማም ዓባይ ሰዓል ኣለውኻ። መብዛሕትኡ ግዜ፡ ንኻልኦት ሰባት ከተመሓላልፎ ትኽእል። ሓኪም፡ ብሓገዝ መድሃኒት ክተሕውየካ እያ።

ፍወሳ መልከፍቲ ባክተርያ ዓባይ ሰዓል

እንታይ? ከኒና

መዓስ መዓስ? መዓልታዊ

ንኽንደይ ግዜ? 3 ወርሒ

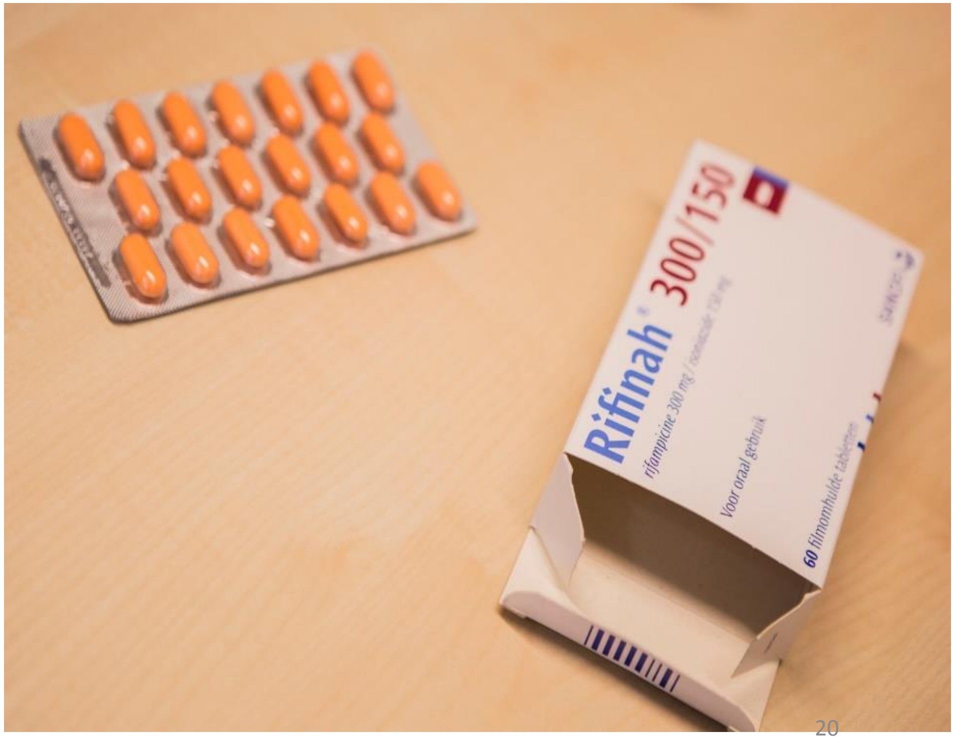

ሕቶ

መልከፍቲ ባክተርያ ዓባይ ሰዓል

ስለምንታይ መርመራ መልከፍቲ ባክተርያ ዓባይ ሰዓል ምግባር የድልየኩም?

- ኣብ ቀርኒ ኣፍሪቃ ሕማም ዓባይ ሰዓል ገና ብብዝሒ ይርከብ
- ኣብ ሃገረ ኔዘርላንድ ዝነብሩ ኤርትራውያን ሕማም ዓባይ ሰዓል ብብዝሒ የማዕብሉ

- ናይ ሳንቡእ ስእሊ (X-ray) ናይ ሳንቡእ ዓባይ ሰዓል ጥራይ'ዩ ከለሊ ዝኽእል
- ባክተርያ ዓባይ ሰዓል ንነዊሕ ግዜ ደቂሱ ክጸንሕ ይኽእል (ንልዕሊ 10 ዓመት)
- እዚ ዝደቀሰ ባክተርያ ብመርመራ ጥራይ'ዩ ክረጋገጽ ዝኽእል

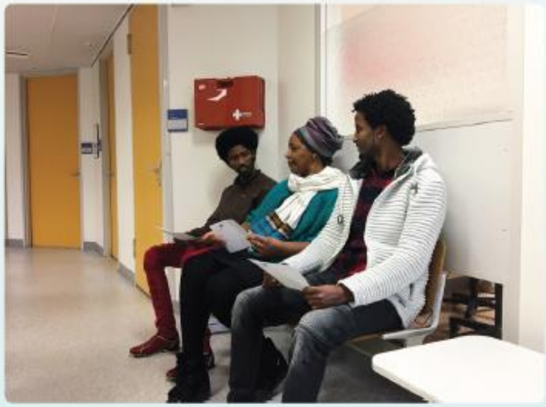

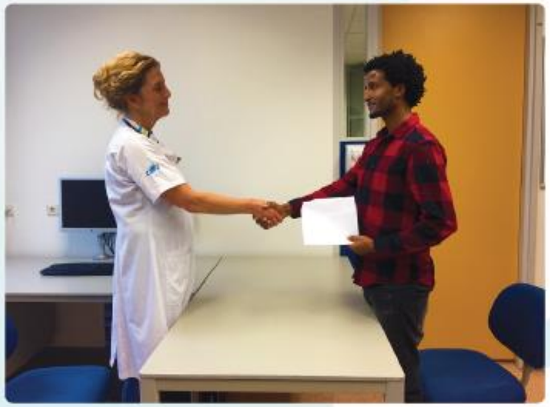

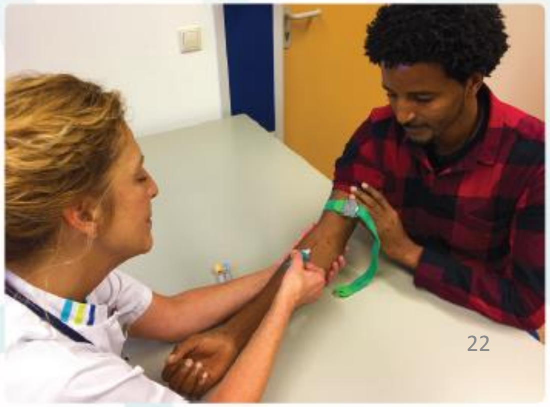

ኣብ ቀረባ መጻኢ ዝካየድ መርመራ ዓባይ ሰዓል

መጽናዕታዊ ፕሮጀክት TB ENDPoint ፍልይ ዝበለ ዕድል ፈጢሩ ይርከብ

- ኣብ ከባቢኹም ዝርከብ ኼ ኼ ደ ዘዓለዎ
- ናይ ናጻ መርመራ
- ናይ ናጻ ፍወሳ

ቤተሰብኩም ወይ መሓዙትኩም ተማልኡ!

ኩሉም ኣብ ኤርትራ ወይ ኢትዮጵያ ዝተወልዱን ኣብ ሃገረ ኔዘርላንድ ትሕቲ 10 ዓመት ዝገበሩን ክሳተፉ ዕዱማት'ዮም!

ናይ መንነት ወረቐትኩምን ናይ መድሕን ካርድኹም ምሳኹም ተማልኡ!
