# Supplementary material for: Knowledge, attitudes, beliefs, and stigma related to latent tuberculosis infection: a qualitative study among Eritreans in the Netherlands
Source: BMC Public Health. 2020 Oct 23;20:1602. doi: 10.1186/s12889-020-09697-z (PMC7585221; doi:10.1186/s12889-020-09697-z)
Supplement: Supplementary file 3 — Additional file 3. Interview topic guides for group and individual interviews with Eritrean asylum seekers (chapter 1) and refugees (chapter 2). [file 12889_2020_9697_MOESM3_ESM.pdf]

Additional file 2
Interview topic guides

Content

1. Interview topic guide group interviews among Eritrean asylum seekers
2. Interview topic guide individual interviews among Eritrean asylum seekers
3. Interview topic guide group interviews among Eritrean refugees
4. Interview topic guide individual interviews among Eritrean refugees

Chapter 1.
Interview topic guide group interviews among Eritrean asylum seekers

Knowledge about tuberculosis

1. Could you explain what the disease tuberculosis is?
   a. Could you explain how you can get the disease tuberculosis?
   If any knowledge exists:
   b. Where did you learn about TB?
2. Do you know anyone from the past who suffered tuberculosis?
   If yes:
   a. What do you remember from this experience?
3. Could you explain the difference between the disease tuberculosis and latent tuberculosis infection?
4. Where did you gain your knowledge about TB?

Information about the LTBI screening

5. Did you receive information about the purpose of tuberculosis infection screening today?
   a. How did you receive information? (oral / print / video)
   b. Was the information you received clear / Did you understand the information?
   c. Would you have liked to receive the information in a different way? If yes, in what way?
   d. What information did you miss or misunderstand?
6. Did you receive an information brochure together with the invitation for the screening?
   If yes:
   a. Did you read the information brochure?
   b. What did you think of the information brochure?
7. Do you think you were well informed about what to expect today and how the screening was organized?

Understanding of the LTBI screening

8. Did you know beforehand what would happen during the tuberculosis infection screening?
   a. What did were your expectations of the screening beforehand?
   b. Did the LTBI screening live up to your expectations beforehand?
9. Can you explain why the screening for tuberculosis infection is organized?
10. Did you understand the information and instruction given by the health staff?

11. Did you feel treated with regard and due respect? Did you have to wait long?
12. Did you feel at ease / comfortable?
13. How did you experience the blood collection?
    a. Was the collection of blood a reason for you to decide to participate or not?

What happens after the screening

14. Can you tell me what the outcomes of the screening / blood test can be?
15. If the blood test shows you are infected with the tuberculosis bacteria, do you know what the follow-up / next steps will be?
16. Do you think the outcome of the screening will / could have any influence on your request for asylum or residence permit?

Stigma and screening for (latent) TB

17. Not all population groups in your center are invited for this LTBI screening, can you explain why?
18. Do you feel comfortable talking about the tuberculosis screening with other people?
    If yes:
    a. What would you discuss?
    b. With whom?
    If no:
    c. Why not?
19. Do you feel concerned about confidentiality in a group screening?
20. Normally the screening takes place at the MHS? What do you think about the screening being organized at the Reception Centre for asylum seekers?
21. How do other people react to the screening?
    a. How do you feel about their reaction?
22. Do you feel free to cooperate with the screening?
23. If you would be diagnosed with TB infection:
    a. Would you tell anyone / talk about it?

Final question

24. If you could improve the screening process for tuberculosis infection, what would you change/ do different in the organization / approach?

**If participants mention the screening for other diseases, then elaborate on that by asking them whether they would have liked to be screened for the other diseases too, which diseases, and why.*

Chapter 2.

Interview topic guide individual interviews among Eritrean asylum seekers

Introduction questions

1. Can you explain what you are/were being treated for?
2. Can you tell us how you have experienced the preventive treatment so far?
    a. Did you experience any problems during the treatment? If so, what were the problems?
    b. What did you like about the treatment?
    c. What did you not like about the treatment?
3. Can you tell us if you think the treatment is important / necessary? Why?

Treatment acceptance

4. Can you explain why you are being treated for latent tuberculosis infection?
5. Can you tell me what kind of information you received about the preventive treatment?
    a. Who gave you the information and how?
    b. Did you understand all the information provided? / Would you like to have received other or additional information?
6. Can you tell me something about the duration of the treatment and the amount of pills?
    a. Can you tell me for how long you had to take the medication?

Treatment compliance

7. Can you tell me why it is important to take the pills every day?
    a. Do you know the consequence if you do not take your pills every day?
8. Did you find it difficult to take the pills every day?
    a. What moments were difficult? Why?
9. Did you sometimes forget to take your pills?
    a. What did you do when you forgot to take your pills?
    b. Can you tell me how you felt when you forgot to take your pills?
    c. Can you tell me how many times you have forgotten your pills?
    d. Have you talked about it with your doctor or your nurse?

        If yes:

        i. What was their response?
        ii. Did you feel comfortable with their response?

        If not:

        i. Why not?
10. Were there moments when you wanted to quit the preventive treatment?

    If yes:

    a. Why did you wanted to quit the treatment?
    b. What did you do when you wanted to quit the preventive treatment?
    c. What kind of help/guidance did you receive from your nurse/doctor when you wanted to quit the preventive treatment?
11. What helped to continue the treatment?
12. Was there anything in particular that motivates you to finish/continue your treatment?

Side effects

13. Did you receive information about potential side effects of the treatment?

    If yes:

    a. What kind of information did you receive?
    b. When did you receive this information?
    c. Who gave you the information?
    d. Did you understand the information?

    If no:

    a. Were you aware of any potential side-effects when you started the treatment?
14. Did you experience any side effects from the treatment?

    If yes:

    a. What kind of side-effects did you experience?
    b. What did you do when you experienced the side-effects?
    c. What kind of guidance/help did you receive from the MHS when you experienced the side-effects?

Consultation MHS staff

15. Can you tell us about the support that the nurses and the doctor gave during the preventive treatment?

    a. How often have you been in contact with the nurse / doctor?
    b. How did you have contact? Telephone / visit MHS / house visit by nurse?
    c. What did you think about this contact?
    d. Did you mostly understand what was told during the contact with the nurse / doctor?
    e. Was there enough time to discuss your questions with the doctor / nurse?

16. How did you travel to the MHS?
    a. How often did you have to travel to the MHS?
    b. Was the MHS easy to find the first time you visited the MHS?
    c. Was it easy to travel to the MHS (regarding time and transitions (bus, train etc.))?
    d. Did you have to pay the travel costs or any other costs yourself?
       If yes:
       i. What costs, and how much were the costs?
       ii. Were the costs acceptable / affordable?

Communication

17. How did you communicate with your doctor / nurse?
18. What did you think about communicating using a interpreter?
    a. Would you rather communicate in a different way (then using a translator via the telephone)?
       If yes:
       i. What way?
19. Did you feel comfortable asking questions to you doctor / nurse?
    If not:
    a. Why not?
    b. What kind of questions did you not ask for example?

Support during treatment

20. Did you receive any support from other people than the MHS staff during your treatment?
    If yes:
    a. From whom?
    b. What kind of support?
    c. How did you like that?
    d. Was it essential support to help you finish the treatment?
    If not:
    e. Would you have wished that there was other or extra support?
    f. What kind of support?

Other

21. How does your social network react to your preventive treatment?
    a. Did you tell your friends or family about the preventive treatment?
       If yes:
       i. How did they react?
       If not:
       ii. Why did you not tell them?
22. Do you have any suggestions how the delivery of screening / preventive treatment can be improved?

Chapter 3

Interview topic guide group interviews among Eritrean refugees

Communication of the intervention

1. How did you hear about the education session about tuberculosis?
   Who told you about the health education about tuberculosis?
2. What do you think about the way you were approach for the health education session?
   Do you think it could have been done in a different or better way? Explain.
   Do you think another method would have been better in reaching more Eritrean migrants?
3. Can you describe your first reaction to the invitation to come to the health education about tuberculosis?
4. What did you think about the fact that the education session was especially designed for Eritrea migrants?

Health education

1. What made you decide to go to the health education about tuberculosis?
2. What did you expect from the health education about tuberculosis?
3. Do you think you have better knowledge now about tuberculosis? Explain.
4. Could you explain what the disease tuberculosis is?
   a. Could you explain how you can get the disease tuberculosis?
5. Could you explain the difference between the disease tuberculosis and latent tuberculosis infection?
6. We can always improve the health education, what do you think can be improved?
7. Did you discuss the health education with other people? What did you discuss?

LTBI screening

1. What made you decide to go to the LTBI screening?
2. What was your reaction to the possibility to get the free screening for LTBI?
3. Did you tell other people about the opportunity to get the LTBI screening? How did they react?
4. What did you expect of the LTBI screening today?
   Do you think you were well informed about what to expect today and how the screening was organized?
5. Do you know why the LTBI screening today was organized? Explain.
6. Do you know what the outcomes of the screening / blood test can be? Explain.
7. If the blood test shows you are infected with the tuberculosis bacteria, do you know what the follow-up / next steps will be?

Stigma and screening for (latent) TB

25. Do you feel comfortable talking about the tuberculosis screening with other people?
    <u>*If yes:*</u>
    a. What would you discuss?
    b. With whom?
    <u>*If no:*</u>
    c. Why not? What would help you?
26. Do you feel concerned about confidentiality in a group screening?
27. How do you think other people would react if you tell them about the LTBI screening?
    a. How do you feel about their reaction?
28. Do you feel free to cooperate with the screening?
29. Do you feel free to receive written information about TB / screening? (or rather hide this from others). Or look at the internet for information freely (also when others can see this?)

Chapter 4
Interview topic guide individual interviews among Eritrean refugees

Positive LTBI test result and initiation of preventive treatment

23. What was your reaction when you read in the letter that you had a positive blood test?
24. Did you share the result of the test with anyone?
25. The letter invited you for an appointment with the doctor. Can you explain how your first consultation with the doctor went?
    a. What did they tell you about your results of the blood test?
    b. What did they tell you about tuberculosis and LTBI?
    c. What did they tell you about the treatment?
26. Did the doctor give you any other information material about LTBI and the treatment?
    a. For example: an information brochure?
27. How did you feel after your first consultation with the doctor?
    a. Could you remember all the information that you were told?
28. How did you feel when you were about to start the treatment?
    a. Did you feel confident?
29. When did you start your treatment?
30. You had to get your medication from the pharmacy. How did that go?

Consultation TB care staff

1. Did you discuss with your TB nurse how they could best assist you during your treatment? Explain.
2. Can you tell us about the support that the nurses and the doctor gave during the preventive treatment?
    b. What do you think of the support that you received?
    c. Did you have any saying in the type of support that you got? Was the support during the treatment mutually discussed?
1. How often have you been in contact with the nurse / doctor?
    a. How did you have contact? Telephone / visit MHS / house visit by nurse?
    b. What did you think about this contact?
    c. Did you understand what was told during the contact with the nurse / doctor?
    d. Was there enough time to discuss your questions with the doctor / nurse?
2. How did you travel to the MHS?
    a. How often did you have to travel to the MHS?
    b. Was the MHS easy to find the first time you visited the MHS?
    c. Was it easy to travel to the MHS (regarding time and transitions (bus, train etc.))?
    d. Did you have to pay the travel costs or any other costs yourself?

Support during treatment

1. Did you tell anyone about your preventive treatment?
    d. Who? How did they react? Were they supportive? How?
2. Did you receive any support from other people than the MHS staff during your treatment?
    If yes:
    a. From whom? What kind of support? Did it help you?
    If not:
    b. Would you have wished that there was other or extra support?
    c. What kind of support?

Stigma

1. In your community, how is a person who has LTBI usually regarded/treated?
2. Did you feel comfortable to discuss your LTBI and preventive treatment with other people in your community?
3. How did people react to the fact that you took preventive treatment for tuberculosis infection? / How do you think people would have reacted (if you told them)?

Communication

1. How did you communicate with your doctor / nurse?
2. How did you experience the interaction with the doctor / nurse? Explain.
3. What did you think about communicating using an interpreter?
4. Did you feel comfortable asking questions to you doctor / nurse? Explain.

Treatment compliance

1. Can you explain why you started treatment for latent tuberculosis infection?
   Can you explain why you did not start treatment for latent tuberculosis infection?
2. Can you tell me something about the duration of the treatment and the amount of pills?
    - b. What do you think about the duration of the treatment?
    - c. Why takes the treatment 3 months?
3. Did the TB nurse give you any advice or tips on how to remember to take your medication?
    - a. What advice / tips did you receive?
    - b. Did it help you?
    - c. Did you come up with tricks yourself to remind yourself to take your medication?
4. Can you explain why it is important to take the pills every day?
5. Why was it so important for you to finish your treatment?
6. Did you find it difficult to take the pills every day?
    - a. What moments were difficult? Why?
7. Did you sometimes forget to take your pills?
    - a. What did you do when you forgot to take your pills?
    - b. Can you tell me how you felt when you forgot to take your pills?
    - c. Can you tell me how many times you have forgotten your pills?
    - d. Have you talked about it with your doctor or your nurse?
8. Were there moments when you wanted to quit the preventive treatment?
    - *If no:*
    - *a. What kept you going?*
    - *If yes:*
    - b. Why did you wanted to quit the treatment?
    - c. What did you do when you wanted to quit the preventive treatment?

Side effects

1. Did you receive information about potential side effects of the treatment? Explain.
2. Did you experience any side effects from the treatment? Explain.
